# Gut microbiota responses to bariatric surgery are associated with metabolic outcomes and type 2 diabetes remission

**DOI:** 10.1038/s42255-026-01525-9

**Published:** 2026-05-07

**Authors:** Lisa M. Olsson, Heidi Borgeraas, Rima M. Chakaroun, Dag Hofsø, Jens Kristoffer Hertel, Chinmay Dwibedi, Matthias Mitteregger, Jens Juul Holst, Valentina Tremaroli, Jøran Hjelmesæth, Fredrik Bäckhed

**Affiliations:** 1 The Wallenberg Laboratory, Department of Molecular and Clinical Medicine, Institute of Medicine, Sahlgrenska Academy, University of Gothenburg, Gothenburg, Sweden; 2 Department of Endocrinology, Obesity and Nutrition, Vestfold Hospital Trust, Tønsberg, Norway; 3 Medical Department III – Endocrinology, Nephrology, Rheumatology, University of Leipzig Medical Center, Leipzig, Germany; 4 Department of Clinical Microbiology and Molecular Infection Medicine Sweden (MIMS), Scilifelab, Umeå University, Umeå, Sweden; 5 Novo Nordisk Foundation Center for Basic Metabolic Research and Department of Biomedical Sciences, Faculty of Health and Medical Sciences, University of Copenhagen, Copenhagen, Denmark; 6 Department of Endocrinology, Morbid Obesity and Preventive Medicine, Institute of Clinical Medicine, University of Oslo, Oslo, Norway; 7 Region Västra Götaland, Sahlgrenska University Hospital, Department of Clinical Physiology, Gothenburg, Sweden; 8 Novo Nordisk Foundation Microbiome Health Initiative and the National Food Institute, Technical University of Denmark, Kongens Lyngby, Denmark

**Keywords:** Endocrine system and metabolic diseases, Microbial communities

## Abstract

Bariatric surgeries, such as Roux-en-Y gastric bypass (RYGB) and sleeve gastrectomy (SG), improve obesity and type 2 diabetes (T2D). Both surgeries affect the gut microbiota, but their contribution to T2D remission remains unclear. In this subanalysis (RYGB, *n* = 39; SG, *n* = 38) of the randomized controlled Oseberg trial (NCT01778738), in which participants underwent either RYGB or SG surgery, we profiled the faecal microbiome of individuals with obesity and T2D before and 12 months after surgery. We show that both surgeries altered the microbiome in the same direction, but with larger changes after RYGB. The SG-associated altered microbiome composition correlated positively with circulating glucagon-like peptide 1 levels, beta-cell function and 5 year T2D remission. Remission was also linked to increased gene richness and metabolic potential for fermentation, methanogenesis and butyrate production. Notably, these associations persisted after accounting for the extent of weight loss. Our findings indicate that surgery-specific microbial adaptations influence metabolic improvements and may help to explain heterogeneity in T2D remission after bariatric surgery.

## Abstract (web summary)

Faecal microbiome profiling of individuals with obesity and type 2 diabetes (T2D) before and 12 months after bariatric surgery links surgery-induced changes in microbiota composition to metabolic improvements and potentially to T2D remission.

## Main

Bariatric surgery is an effective treatment for severe obesity and co-morbidities such as type 2 diabetes (T2D)^1,2^. However, long-term weight loss is heterogeneous among individuals following different procedures^3^, and the metabolic improvements underlying T2D remission are only partially explained by weight loss^4^. The gut microbiota, the collective term for the microorganisms that inhabit the gut, is altered after surgery and has been suggested to contribute to metabolic improvements in the host^5–9^. The gut microbiota ferments indigestible dietary components, leading to the production of metabolites, for example, short-chain fatty acids (SCFAs), which provide energy and act as signalling molecules in the host^10^. Bacterial gene richness and SCFA-producing bacteria, especially butyrate producers, are reduced in the gut microbiota of individuals with obesity and T2D^11,12^. Accordingly, increased gene richness and abundance of SCFA-producing bacteria correlate with metabolic health^13,14^ and also improved glucose control in patients with T2D following dietary interventions^15^. Furthermore, SCFAs and biosynthetic pathways for butyrate production have been causally linked to insulin secretion, postprandial glycemic responses and T2D risk^13,14^. Therefore, these features may represent microbial contributions to the outcomes of bariatric surgery relevant for T2D management.

The type of surgical procedure appears to have an impact on the extent of metabolic improvements after bariatric surgery. In a randomized clinical trial in which the patients, attending physician and statistician were blinded to the surgical procedure (the Oseberg study), we showed that RYGB was superior to SG, both in terms of weight loss and T2D remission up to 5 years after surgery^16–18^. There are significant differences in the anatomical rearrangements resulting from these procedures, possibly contributing to the differences in microbiome alterations previously observed when comparing RYGB and SG^19–21^. However, these earlier studies were not designed to investigate the impact of different surgeries on the gut microbiome and its potential link to T2D remission.

To investigate the effects of RYGB and SG on the microbiome and assess whether surgery-induced alterations were associated with T2D remission, we characterized the faecal microbiome of 77 individuals with obesity and T2D from the Oseberg study^17^. Microbiome profiling was performed by whole-genome sequencing at baseline and 12 months postoperatively.

## Results

### Clinical characteristics before and 12 months after surgery

The individuals included in this study are a subset of participants from the Oseberg trial who had provided faecal samples both before and 12 months after RYGB (*n* = 39) or SG (*n* = 38). Clinical characteristics at baseline did not differ between the individuals randomized to either surgery (Supplementary Table 1).

**Fig. 1:**
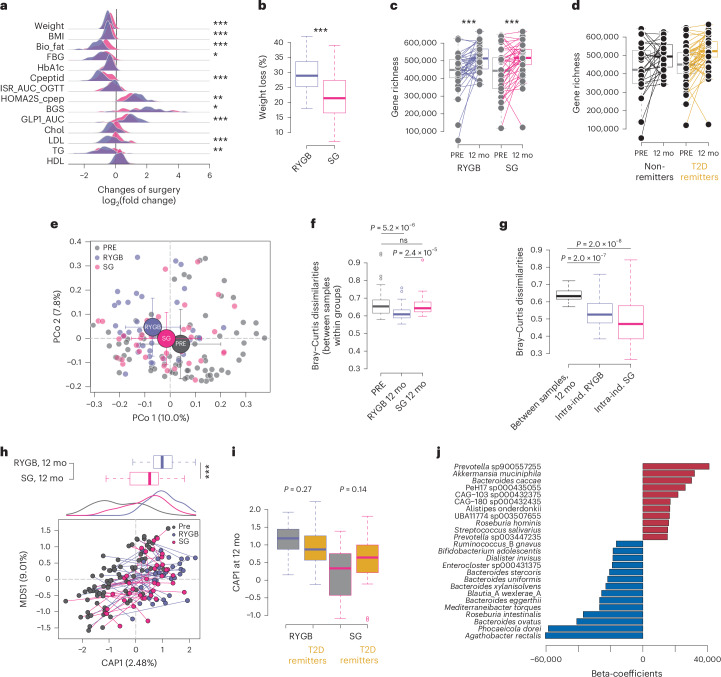
Microbiome composition after bariatric surgery is associated with metabolic improvements. **a**, Density plots of log_2_(fold changes) (calculated at 12 months from baseline) for clinical variables in participants with RYGB (purple) or SG (pink). Differences between RYGB and SG were tested using the Wilcoxon rank-sum test: *P_adj_ < 0.05; **P_adj_ < 0.01; ***P_adj_ < 0.001. **b**, Boxplots of weight loss calculated as percentage of baseline weight (*P* = 0.0003, Wilcoxon rank-sum test). **c**, Microbiome gene richness at baseline (PRE) and 12 months after surgery (12 mo) in RYGB and SG; lines connect samples from the same individual (*P* = 0.0004 for RYGB, *P* = 0.001 for SG; Wilcoxon signed-rank test). **d**, Microbiome gene richness at baseline and 12 mo for individuals with T2D remission (yellow) or no remission (black); lines connect samples from the same individual. The effect of T2D remission on the increase in gene richness was significantly larger than the effect of surgery (LongDat; see Methods). **e**, Dimension reduction of species composition of baseline and 12 mo samples using principal component (PCo) analysis on Bray–Curtis dissimilarity; shown are PCo 1 and 2. Large circles indicate group average values, and arrows indicate the standard deviation from the mean. **f**, Boxplots of average inter-individual Bray–Curtis dissimilarity (one value per sample) calculated for baseline and 12 mo samples (RYGB 12 mo and SG 12 mo). ns, not significant. **g**, Boxplots of average inter-individual Bray–Curtis dissimilarity calculated for 12 mo samples (one value per sample), and intra-individual Bray–Curtis dissimilarity calculated between samples from each individual (baseline versus 12 mo) in the RYGB and SG groups. **h**, Constrained analysis of principal coordinates using sampling time (PRE and 12 mo) as constraining variable on Bray–Curtis dissimilarity. First component of constrained ordination (CAP1) and first multidimensional scaling variable (MDS1) are plotted; lines connect samples from the same individual. Top, silhouettes represent density plots for CAP1 in baseline samples (grey) and 12 mo samples for RYGB (purple) and SG (pink); boxplots represent CAP1 in 12 mo samples for the RYGB and SG groups (*P* = 0.0002, for weight-loss-adjusted linear model). **i**, Boxplots for CAP1 at 12 mo in participants with RYGB or SG, divided according to T2D remission. **j**. Bar plot of beta coefficients for the top 25 species contributing to CAP1. Boxplots show the median (centre), interquartile range (IQR; 25th–75th percentiles) and whiskers extending to 1.5× IQR. *P* values are from two-sided Wilcoxon signed-rank tests unless otherwise specified. RYGB, *n* = 39 participants; SG, *n* = 38 participants, each with paired samples at baseline and 12 months. Bio_fat, fat mass; FBG, fasting blood glucose; Cpeptid, connecting peptide; ISR_AUC_OGTT, insulin secretion rate, area under the curve, during oral glucose tolerance test; Chol, total cholesterol; LDL, low density lipoprotein, TG, triglycerides; HDL, high density lipoprotein. Source data

Both surgeries resulted in significant weight loss and improved metabolic health 12 months after surgery, but there were differences in the extent of improvement between the groups (Supplementary Table 1 and Fig. 1a), consistent with the results observed in the full cohort^17^. Compared with individuals who underwent SG, participants who underwent RYGB lost more weight (Fig. 1a,b) and had greater reductions of fat mass, low-density lipoprotein and total cholesterol levels (Supplementary Table 1 and Fig. 1a). Furthermore, we observed a greater reduction in fasting blood glucose after RYGB compared to SG, together with greater increase in insulin secretion (β cell glucose sensitivity (BGS)) and insulin sensitivity (HOMA2S), and higher circulating levels of glucagon-like peptide 1 (GLP-1) after an oral glucose load (area under the curve GLP-1 and 15 min GLP-1) (Supplementary Table 1 and Fig. 1a). Insulin sensitivity (MinmodSI) and the acute insulin response to glucose (AIRg) measured using an intravenous glucose tolerance test improved after both surgery types, but these improvements did not differ between the groups (Supplementary Table 1). Consistent with the larger improvements in metabolic markers, the T2D remission rate at 12 month follow-up was higher following RYGB than after SG (74% versus 52%; logistic regression, *P* = 0.046). Overall, these results suggest that the superior metabolic improvements after RYGB may involve additional gastrointestinal mechanisms, such as incretin effects.

### RYGB produces larger compositional shifts and a more homogenous gut microbiota

We analysed whole-genome metagenomic data to determine how the gut microbiota changed after RYGB and SG, and in relation to T2D remission. In agreement with earlier studies^22^, gut microbiota gene richness increased after both surgery types (Fig. 1c), and the increase was larger in individuals who were in remission from T2D at the 12 month follow-up (Fig. 1d).

The composition of the gut microbiota at baseline did not differ between the groups randomized to RYGB or SG (permutational MANOVA, *P* = 0.25). Gut microbiota profiles 12 months after RYGB or SG were concordant along the first two principal coordinates (Fig. 1e), suggesting similar effects of the two procedures on the overall composition. However, RYGB explained 5.3% of the gut microbiota compositional variation (permutational MANOVA, *P* = 0.0001) while SG only explained 1.9% (permutational MANOVA, *P* = 0.045) (Fig. 1e). The larger effect of RYGB remained significant after adjustment for weight loss (*R*^2^ = 0.03, *P* = 0.001).

We also observed lower inter-individual Bray–Curtis dissimilarity after RYGB compared to both baseline and SG (Fig. 1f), indicating a more homogeneous composition after RYGB. However, the average inter-individual variation 12 months after surgery was larger than the intra-individual variation from baseline for both RYGB and SG (Fig. 1g). These results show that the impact of bariatric surgery is smaller than the inter-individual variation, and the gut microbiota remains individualized after surgery, as seen in individuals who have not undergone surgical intervention^23^.

**Extended Data Fig. 1:**
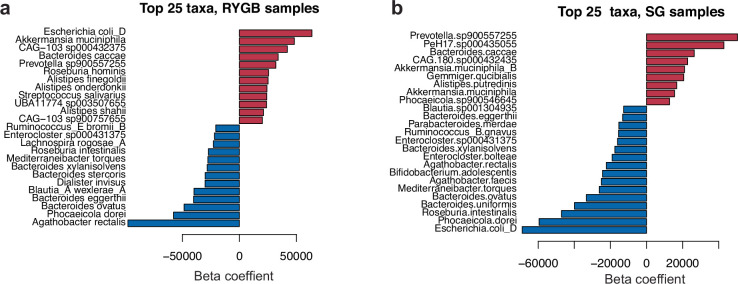
Beta coefficients for CAP1. Bar plots of beta coefficients for the top 25 species contributing to CAP1 **a**. in RYGB and **b**. SG samples.

Analysis of gut microbiota composition using sampling time as a constraining variable showed that surgery explained 2.5% of the overall compositional variation (permutational MANOVA, *P* = 1 × 10^−4^) (Fig. 1h). The surgery-induced change based on the first component of constrained ordination (CAP1) was greater after RYGB than after SG, even after adjustment for weight loss (linear model, *P* = 0.0001), and was more homogenous after RYGB (Fig. 1h, boxplots). We also observed that CAP1 was higher, albeit non-significantly, after SG but not after RYGB in individuals with T2D remission compared with those not in remission (Fig. 1i). Species linked to high CAP1 included *Akkermansia muciniphila* and uncharacterized species from *Prevotella* (*Prevotella* sp900557255), Oscillospirales (CAG-180 sp000432435, CAG-103 sp000432375), Christensenellales (PeH17 sp000435055) and Lachnospiraceae (UBA11774 sp003507655)^24^. By contrast, species linked to low CAP1 included *Agathobacter rectalis* (previously known as *Eubacterium rectale*), several species in *Bacteroides*, *Ruminococcus*_B *gnavus* and *Mediterraneibacter torques* (previously known as *Ruminococcus torques*) (Fig. 1j). We repeated these analyses within each surgery group and observed that the top 25 species linked to CAP1 were similar after either surgery. However, *Escherichia coli* correlated positively with CAP1 after RYGB but negatively after SG (Extended Data Fig. 1a,b).

#### Gut microbiota profiles correlate with metabolic improvements, particularly after SG

**Fig. 2:**
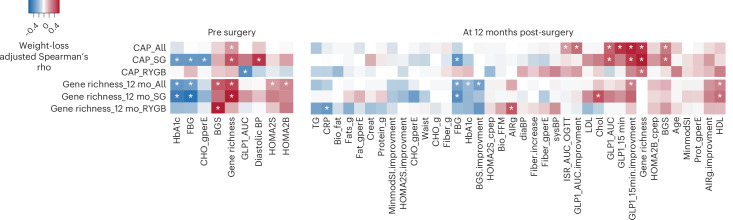
Shift in fermentation following bariatric surgery. Heatmap of weight-loss-adjusted Spearman correlations assessed for CAP1 and gene richness at 12 mo against variables at baseline (left) or at the 12 month follow-up (right). For the 12 month follow-up, the plot contains both measured values at 12 months and changes from baseline (indicated as improvements) as well as dietary data in both grams (g) and energy normalized (gperE). *P < 0.05. RYGB, *n* = 39 participants; SG, *n* = 38 participants, each with paired samples at baseline and 12 months. CHO_gperE, carbohydrates intake, gram per energy; fat, fat intake; creat, creatinine; FFM, fat free mass; diaBP, diastolic blood pressure; sysBP, systolic blood pressure. Source data

Surgery-induced microbiota change (CAP1) correlated positively with baseline gene richness and negatively with baseline hemoglobin A1c (HbA1c), fasting blood glucose and carbohydrate intake, normalized by total energy, in participants randomized to SG but not in those randomized to RYGB (Fig. 2a). In SG, baseline gene richness was associated with both higher gene richness at 12 months as well as larger CAP1, while it correlated positively only with insulin secretion (BGS) in participants randomized to RYGB.

Following the more heterogeneous microbiome shifts and greater variation between individuals after SG, CAP1 showed positive correlations at the 12 month follow-up with gene richness, BGS and GLP-1 responses, particularly after SG (Fig. 2a). Gene richness at 12 months correlated negatively with fasting blood glucose and HbA1c in the full cohort and with C-reactive protein (CRP) in participants with RYGB. Positive correlations were observed with improved GLP-1 responses at 12 months in the full cohort (Fig. 2a). Insulin secretion (AIRg) and insulin sensitivity (MinmodSI) assessed intravenously at 12 months did not correlate with CAP1 or gene richness at 12 months in the full cohort or in participants with SG, but a positive correlation was observed between gene richness at 12 months and AIRg in participants with RYGB (Fig. 2a). In SG, gene richness at the 12 month follow-up correlated negatively with baseline HbA1c and fasting blood glucose and lower fasting blood glucose after surgery (Fig. 2a). Neither CAP1 nor gene richness at 12 months correlated with postoperative dietary intake, whether expressed in absolute grams or normalized to energy intake, in either RYGB or SG (Fig. 2a).

These results show that a specific gut microbiota composition, represented by high CAP1, and high gene richness 12 months after surgery was more likely to be observed in individuals with more controlled glucose metabolism and high gene richness at baseline, as well as in individuals with greater GLP-1 and insulin responses after an oral glucose load and better glycemic control after surgery. In most cases, the correlations were stronger in those randomized to SG (Fig. 2a), the group with less pronounced and more heterogeneous metabolic improvements showing a lower T2D remission rate 12 months after surgery (Fig. 1a and Supplementary Table 1).

#### Species altered by RYGB and SG

Next, we assessed how RYGB and SG affected the abundance of gut microbial species. As several common drugs affect gut microbiota composition^25^, and medication use changed after surgery (Supplementary Table 1), we filtered out features that covaried with change in use of statins, antihypertensive medications and proton pump inhibitors. Metformin use covaried strongly with remission status and therefore could not be controlled for. Additionally, given that we observed different weight loss after RYGB and SG (Fig. 1a,b), and as weight loss might drive microbiome changes, we also examined which species-level changes covaried with changes in weight, body composition and metabolic parameters, and whether these relationships differed from associations with T2D remission.

**Fig. 3:**
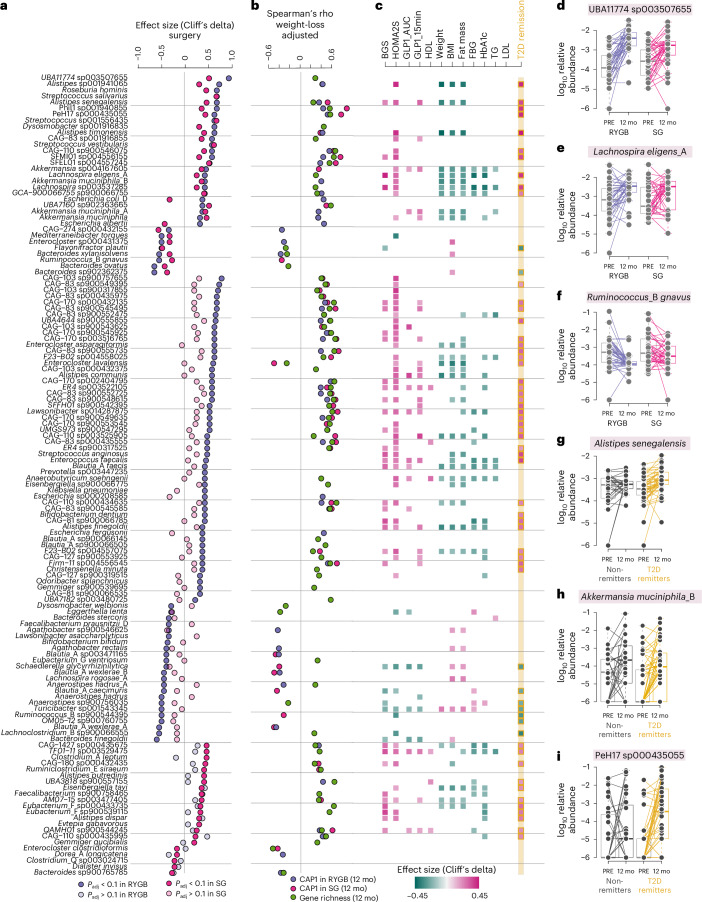
Species altered by RYGB and SG. **a**, Species significantly altered after RYGB and SG (*P*_adj _< 0.05, Wilcoxon signed-rank test) with an average count of more than 6,500 or covarying with remission. Additional statistics are provided in Supplementary Table 2. Points show effect sizes (Cliff’s delta) for the change following surgery (RYGB, purple; SG, pink). **b**, Significant weight-loss-adjusted Spearman’s rho correlations (*P* < 0.05) for species abundances with gene richness at 12 mo (green) and CAP1 in 12 mo samples for RYGB (purple) and SG (pink). **c**, Effect sizes (Cliff’s delta) for covariates classified as either ‘entangled with covariate’ or ‘reducible to covariate’ in explaining species changes following surgery (LongDat). **d**–**f**, log_10_ relative abundance of selected species at baseline and 12 mo after surgery in RYGB and SG; lines connect samples from the same individual. **g**–**i**, log_10_ relative abundance of selected species at baseline and 12 mo after surgery in participants divided by T2D remission at 12 mo. Lines connect samples from the same individual. Boxplots show the median (centre), IQR (25th–75th percentiles) and whiskers extending to 1.5× IQR. *P* values are from two-sided Wilcoxon signed-rank tests unless otherwise specified. RYGB, *n* = 39 participants; SG, *n* = 38 participants, each with paired samples at baseline and 12 months. Source data

Species that increased significantly in abundance after either surgery type included uncharacterized Lachnospiraceae (UBA11774 sp003507655), Christensenellales and Oscillospiraceae, as well as *Alistipes* spp., *Streptococcus* spp., *Akkermansia* spp. and *Lachnospira eligens* (previously known as *Eubacterium eligens*) (Fig. 3a,d,e and Supplementary Table 2). Species that decreased significantly in abundance after either surgery type included *M.* *torques* and *R.* *gnavus*, which have been linked to intestinal inflammation, metabolic syndrome and T2D^11,26^, as well as species known to metabolize complex carbohydrates, such as *Bacteroides* spp. and *Blautia wexlerae* (Fig. 3a,f and Supplementary Table 2).

In line with the more homogenous composition found after RYGB (Fig. 1h), more species changed significantly in abundance after RYGB than after SG (Fig. 3a and Supplementary Table 2). Examples of species that increased significantly after RYGB included abundant, but uncharacterized, Firmicutes only known as metagenome-assembled genomes (such as CAG-170 sp000432135), potential butyrate producers in *Alistipes*, *Christensenella minuta* and the butyrate producer *Anaerobutyricum soehngenii* (Fig. 3a and Supplementary Table 2). The abundance of other common butyrate producers, such as *Faecalibacterium* clade D and *Anaerostipes* spp., and carbohydrate degraders such as *Bifidobacterium* and *Bacteroides* spp. decreased after RYGB (Fig. 3a).

A smaller number of species were significantly altered in abundance only after SG (Fig. 3a and Supplementary Table 2). These included increases in the recently described species *Gemmiger*, an abundant but still poorly characterized Clostridiales phylogenetically placed between *Ruminococcus* and *Faecalibacterium*^27^, and *Eisenbergiella tayi*, a recently isolated non-proteolytic butyrate producer in the Lachnospiraceae family^28^.

In agreement with the similar compositional profiles observed 12 months after surgery between the groups (Fig. 1e), the abundance of most of the species covaried with CAP1 (Fig. 3a and Supplementary Table 2) and with gene richness regardless of surgery type (Fig. 3b and Supplementary Table 2). Only two of the species that changed significantly in abundance after both surgeries shifted in different directions, increasing after RYGB and decreasing after SG (Fig. 3a); these species belonged to the genus *Escherichia* and their abundance at 12 months covaried with CAP1 following RYGB (Fig. 3b).

Finally, we assessed how changes in abundance of the measured species covaried with metabolic variables and T2D remission after 12 months (in both groups combined) (Fig. 3c and Supplementary Table 2). For many species, the change in abundance covaried with weight, parameters of body composition and glucose metabolism as well as GLP-1 responses (Fig. 3c). For most species linked with T2D remission (for example, *Alistipes senegalensis*, *Alistipes timonensis* and *L.* *eligens*), the change in abundance also covaried with metabolic improvements and weight reduction (Fig. 3c,g). For several *Akkermansia* species, change in abundance covaried with weight and fat mass change but not with T2D remission (Fig. 3c,h). Changes in abundance for a few species, which were uncharacterized and mostly belonged to Christensenellales (for example, PeH17 sp000435055), covaried with T2D remission and not with weight or fat mass loss (Fig. 3c,i). Therefore, these results show that most species-level changes covaried with changes in weight and body composition, but some features linked to T2D remission did not. None of these species-level changes covaried with changes in macronutrient intake (Supplementary Table 2).

#### Altered microbiome functional potential after RYGB and SG

**Fig. 4:**
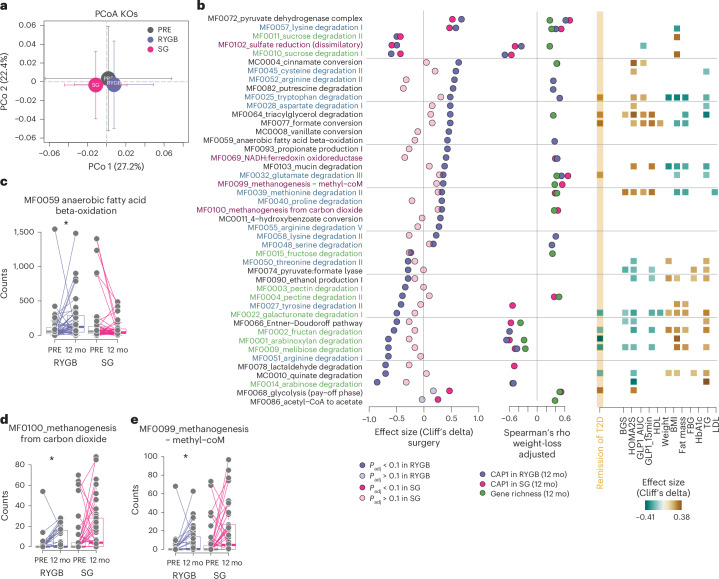
The microbiome functional potential shifted after bariatric surgery. **a**, Dimension reduction of KO composition in baseline and 12 mo samples using PCo analysis on Bray–Curtis dissimilarity; shown are PCo 1 and 2. Circles indicate group average values, and arrows indicate the standard deviation from the mean. **b**, GMMs significantly altered after RYGB and SG. Shown are features with *P*_adj_ < 0.1 (Wilcoxon signed-rank test). Additional statistics are presented in Supplementary Table 4. The first column shows effect sizes (Cliff’s delta) for changes following surgery (RYGB, purple; SG, pink). The second column shows significant weight-loss-adjusted Spearman’s rho correlations (*P* < 0.05) for GMMs with gene richness at 12 mo (green), and CAP1 in 12 mo samples for RYGB (purple) and SG (pink). The third column shows effect sizes for covariates classified as either ‘entangled with covariate’ or ‘reducible to covariate’ in explaining species changes following surgery (LongDat). Colours of GMM names represent ‘amino acid degradation’ (blue), ‘carbohydrate degradation’ (green) and ‘gas metabolism’ (pink). **c–e**, Abundance of selected GMMs at baseline and 12 mo after surgery in RYGB and SG; lines connect samples from the same individual. **c**, MF0059 (RYGB; *P* = 0.03, SG; *P* = 0.40); **d**, MF0100 (RYGB; *P* = 0.019, SG; *P* = 0.27); **e**, MF00099 (RYGB; *P* = 0.017, SG; *P* = 0.18). Boxplots show the median (centre), IQR (25th–75th percentiles) and whiskers extending to 1.5× IQR. *P* values are from two-sided Wilcoxon signed-rank tests unless otherwise specified. RYGB, *n* = 39 participants; SG, *n* = 38 participants, each with paired samples at baseline and 12 months. Source data

By using KEGG orthologs (KOs), we observed that the microbiome functional potential was significantly altered after RYGB (permutational MANOVA on KOs, *R*^2^ = 0.027, *P* = 0.0302) (Fig. 4a and Supplementary Table 3), but not after SG (*R*^2^ = 0.021, *P* = 0.0920). We also observed that the functional potential 12 months after surgery differed significantly between the surgery types (*R*^2^ = 0.026, *P* = 0.0325) (Fig. 4a). To evaluate whether the lack of a significant functional shift after SG was because of heterogeneity in this group, we repeated the analysis in SG participants who achieved T2D remission. In this subgroup, the functional shift was larger and close to significance (*n* = 40, *R*^2^ = 0.043, *P* = 0.055). Similarly, when excluding SG participants with weight loss of less than 10 kg, a significant functional shift was observed (*n* = 66, *R*^2^ = 0.029, *P* = 0.044). Therefore, functional changes after SG occur especially in individuals with greater metabolic improvements, but the heterogeneity of weight loss and T2D remission in the whole SG group dilutes the overall signal.

To identify metabolic pathways affected by surgery, we summarized KOs into gut metabolic modules (GMMs)^29,30^. Abundance of the following GMMs changed in the same direction after RYGB and SG: pyruvate dehydrogenase complex (MF0072, producing acetyl-CoA from pyruvate) and lysine degradation I (MF0057, producing butyrate through the lysine pathway) increased, while sucrose degradation (MF0010, MF0011) and sulfate reduction (MF0102) decreased (Fig. 4b). Pyruvate dehydrogenase complex and lysine degradation I covaried with CAP1 independent of surgery type and gene richness 12 months after surgery (Fig. 4b and Supplementary Table 4).

Abundance of most GMMs, including MF0093 for propionate production, changed significantly only after RYGB (Fig. 4b). Consistent with changes at the species level (Fig. 3a), pathways for metabolism of amino acids and diamines, such as arginine, lysine and putrescine (MF0052, MF0058 and MF0082), widespread in Proteobacteria, were also increased after RYGB, together with modules for oxygen-dependent metabolism, such as ferredoxin oxidoreductase and vanillate conversion (MF0069 and MC0008). Furthermore, anaerobic fatty acid oxidation (MF0059), also widespread in Proteobacteria^31^, increased and covaried with CAP1 only after RYGB (Fig. 4b,c).

**Extended Data Fig. 2:**
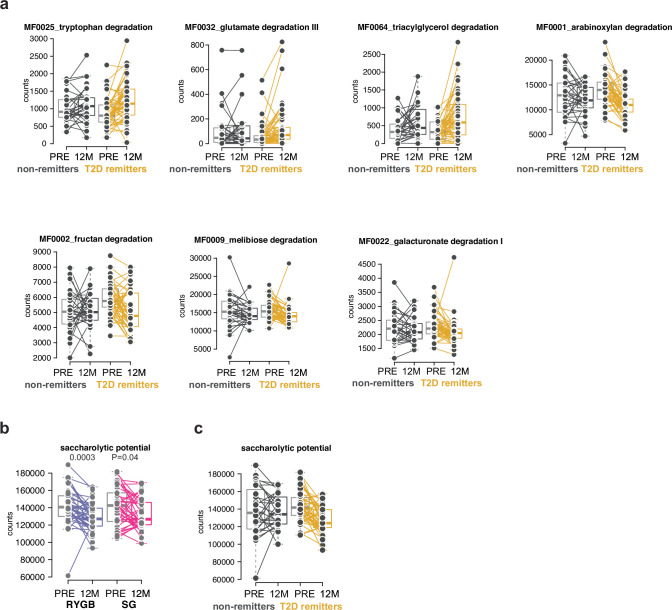
GMMs liked to remission. **a**. Abundance of GMMs at baseline (PRE) and 12 M after surgery in participants divided by T2D remission at 12 months. **b**. Saccharolytic potential at baseline (PRE) and 12 months after surgery (12M) in RYGB and SG. **c**. Saccharolytic potential at baseline (PRE) and 12 months after surgery (12M) in participants divided by T2D remission at 12 months. Boxplots show the median (centre), interquartile range (25th–75th percentiles), and whiskers extending to 1.5×IQR. Dots indicate individual samples and lines connect samples from the same individual. Statistics in Supplementary Table 4. P values are from two-sided Wilcoxon signed-rank tests unless otherwise specified. RYGB, n = 39 participants; SG, n = 38 participants, each with paired samples at baseline and 12 months.

Several GMMs that increased after RYGB also increased after SG, albeit non-significantly. These included pathways for amino acid metabolism to pyruvate, such as MF0025 (tryptophan degradation) and MF0032 (methylaspartate pathway for glutamate catabolism), which were linked to remission (Extended Data Fig. 2a and Supplementary Table 4), as well as MF0045 (cysteine degradation) and MF0048 (serine degradation). MF0064, for triacylglycerol degradation, also had a similar pattern and was linked with remission and gene richness 12 months after surgery (Extended Data Fig. 2a and Supplementary Table 4). In addition, MF0100 and MF0099, the GMMs for methanogenesis from carbon dioxide and hydrogen, which covaried with gene richness 12 months after surgery and with CAP1 after SG (Fig. 4b,d,e and Supplementary Table 4), also increased after surgery. Similarly, MF0103, the GMM for mucin degradation, also increased. The increased capacity for methanogenesis and mucus degradation probably reflects the results at the species level (for example, increased abundance of methanogenic archaea *Methanobrevibacter smithii* and the mucin degrader *A.* *muciniphila*) (Supplementary Table 2).

**Extended Data Fig. 3:**
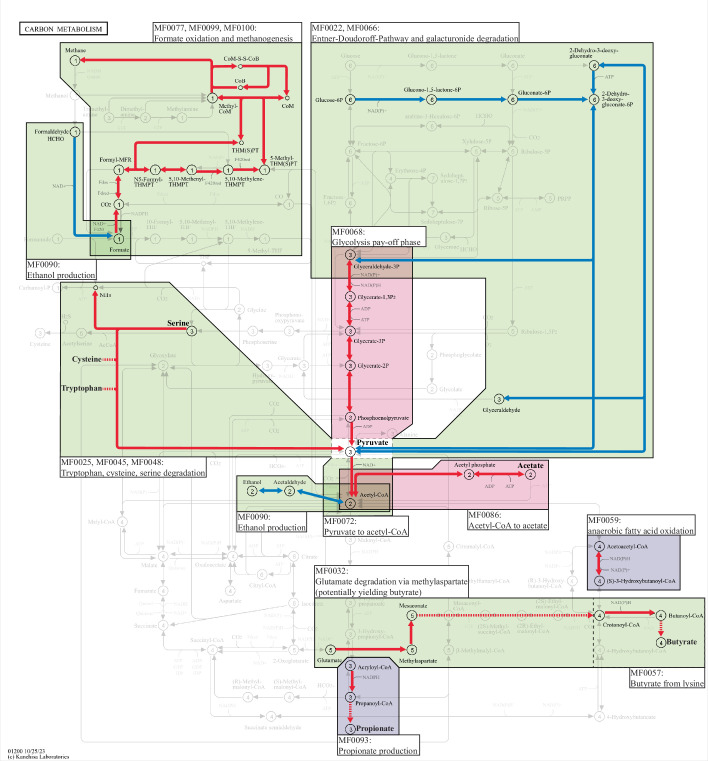
Central carbon metabolism altered in bariatric surgery. Gut metabolic modules (GMMs) for pyruvate and central carbon metabolism, overlaid on the KEGG map for central carbon metabolism (map01200)^76^, decreased (blue arrows) or increased (red arrows) 12 M after bariatric surgery; arrows indicate direction of reactions. Dotted arrows indicate multi-step reactions (cysteine and tryptophan), or additional steps not included in GMMs (butyrate and propionate, drawn according to the metabolic maps described by Louis and Flint^34^). Numbers in circles indicate the number of carbon atoms in metabolites. Box colors indicate if GMMs are significantly decreased or increased in SG only (pink), or in RYGB only (purple) or in either surgery with a consistent direction for change in both surgery types (green). Several GMMs altered after bariatric surgery involve pyruvate metabolism (dashed box). Copyright permission was granted by Kanehisa Laboratories for the use of KEGG pathway map01200.

GMMs with decreased abundance after RYGB included pathways for degradation of monosaccharides, disaccharides and polysaccharides (for example, fructose, MF0015; arabinose, MF0014; melibiose, MF009; arabinoxylan, MF0001; and pectin, MF003); several of these pathways covaried with CAP1 in one or both surgery types and T2D remission (MF0001, MF0002, MF0009) (Fig. 4b, Extended Data Fig. 2a and Supplementary Table 4). Consistent with these results, and possibly related to the decrease of *Bacteroides* and *Bifidobacterium* spp. (Fig. 3a), the saccharolytic potential decreased after both RYGB and SG, particularly in individuals who achieved remission (Extended Data Fig. 2b and Supplementary Table 4). Pathways for pyruvate metabolism to ethanol (MF0090) and formate (MF0074) also decreased after RYGB and non-significantly after SG, together with galacturonate degradation (MF0022) and the Entner–Doudoroff pathway (MF0066) (Extended Data Fig. 3), suggesting decreased potential for non-glycolytic energy metabolism after bariatric surgery^32^.

Finally, the pay-off phase of glycolysis (MF0068) and acetate production from acetyl-CoA (MF0086) increased significantly only after SG and were linked to gene richness, CAP1 and, for MF0068, T2D remission (Fig. 4b and Supplementary Table 4). Overall, these results indicate that the fermentative potential for production of butyrate increased after either surgery type, whereas the potential for production of acetate and propionate increased after SG and RYGB, respectively (Extended Data Fig. 3). Bariatric surgery also increased the potential for metabolism of fatty acids, as indicated by the increase of triacylglycerol degradation (MF0064) and anaerobic fatty acid beta−oxidation (MF0059), but with a larger effect after RYGB. Oxygen-dependent metabolism was specifically increased only after RYGB (MF0069 and MC0008).

#### Altered patterns of carbohydrate and amino acid fermentation after bariatric surgery

Bariatric surgery, particularly RYGB, has been associated with shifts in microbial fermentation patterns, including increased amino acid fermentation^9,33^. To further investigate the fermenting capacity of the microbiome, we determined the faecal levels of SCFAs (acetate, propionate and butyrate), branched-chain fatty acids (BCFAs; isovalerate and isobutyrate), lactate and succinate at baseline and 12 months after surgery.

**Extended Data Fig. 4:**
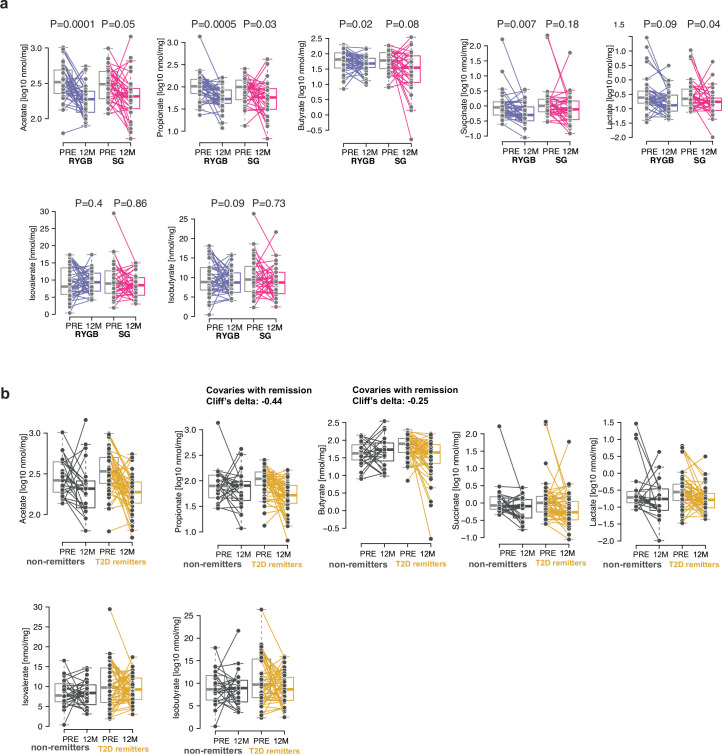
Fecal SCFA and BCFA altered in surgery and linked to remission. Concentrations of short-chain fatty acids (SCFA), branched-chain fatty acids (BCFA), lactate and succinate at baseline (PRE) and 12 months after surgery (12M) in **a**. RYGB and SG, and **b**. samples divided by T2D remission at 12 M. Boxplots show the median (centre), interquartile range (25th–75th percentiles), and whiskers extending to 1.5 × IQR. Dots indicate individual samples and lines connect samples from the same individual. P values are from two-sided Wilcoxon signed-rank tests unless otherwise specified. Covariation test using LongDat (see methods). RYGB, n = 39 participants; SG, n = 38 participants, each with paired samples at baseline and 12 months.

We observed that the increases in functional potential were not reflected in the faecal SCFAs concentrations that decreased 12 months after surgery, while the levels of BCFAs did not change (Extended Data Fig. 4a and Supplementary Table 5). Lactate and succinate are intermediary metabolites that do not accumulate in the human gut^34^, and the lower levels after RYGB possibly suggest increased removal after this surgery. Of note, acetate faecal concentrations decreased in all individuals irrespective of T2D remission, whereas the decrease in propionate and butyrate faecal levels was driven by reduced levels in individuals with T2D remission 12 months after surgery (Extended Data Fig. 4b and Supplementary Table 5).

**Fig. 5:**
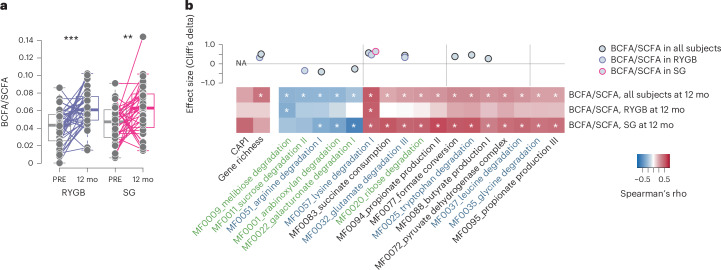
Altered fermentation after bariatric surgery. **a**, BCFA/SCFA ratios at baseline and 12 mo after surgery in RYGB and SG (RYGB, *P* = 0.001; SG, *P* = 0.006). Lines connect samples from the same individual. **b**, Bottom, Spearman’s rho between BCFA/SCFA and GMMs at 12 mo; white asterisks indicate weight-loss-adjusted and false discovery rate-adjusted *P* < 0.1. Top, change in BCFA/SCFA ratio (Cliff’s delta) when the change in ratio between time points covaried with the change of surgery in all samples (LongDat). Boxplots show the median (centre), IQR (25th–75th percentiles) and whiskers extending to 1.5× IQR. *P* values are from two-sided Wilcoxon signed-rank tests unless otherwise specified. RYGB, *n* = 39 participants; SG, *n* = 38 participants, each with paired samples at baseline and 12 months. Additional statistics provided in Supplementary Table 5. Source data

To assess the overall output of microbial fermentations from carbohydrates and proteins, we calculated the ratio of BCFAs to SCFAs (BCFA/SCFA). Consistent with results from cross-sectional studies in RYGB^9,33^, BCFA/SCFA increased after both surgery types (Fig. 5a).

After adjustment for the extent of weight loss, the BCFA/SCFA ratio at 12 months correlated positively with gene richness at 12 months (Fig. 5b, bottom). The change in BCFA/SCFA covaried with shifts in GMMs after surgery (for example, sucrose and galacturonate degradation (MF0011, MF0022) and butyrate production from lysine (MF0057)) (Fig. 5b, top). BCFA/SCFA at 12 months also correlated positively with higher relative abundance of these GMMs and of other pathways for butyrate and SCFAs production (for example, butyrate kinase gene (MF0088), succinate consumption (MF0083) and propionate production (MF0095, MF0094)) (Fig. 5b, bottom). These associations were mostly driven by SG and probably reflected the heterogeneity of microbial shifts after this procedure.

#### Butyrate production potential is increased 12 months after surgery

To specifically quantify the potential for butyrate production and test the link with T2D remission, we quantified the terminal genes for butyrate production from carbohydrates (that is, *but* and *buk*) and amino acids (that is, *ato* for the lysine pathway and *4hbt* for the 4-aminobutyrate pathway)^35^.

**Fig. 6:**
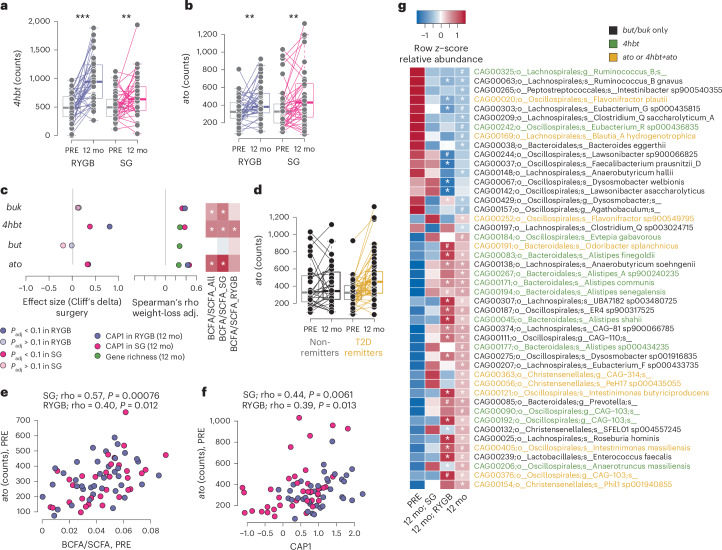
Butyrate production potential increases after bariatric surgery. Counts at baseline and 12 mo after surgery *4hbt* (**a**, 4-aminobutyrate pathway, RYGB; *P* = 0.000007, SG; *P* = 0.02) and *ato* (**b**, lysine pathway; RYGB; *P* = 0.01, SG; *P* = 0.009). Lines connect samples from the same individual. Boxes show median and IQR; whiskers specify ±1.5× IQR. **c**, Change in the abundance of butyrate production terminal genes and correlations with gene richness, CAP1 at 12 mo and the BCFA/SCFA ratio. The first column shows effect sizes (Cliff’s delta) for change following surgery (two-sided) for RYGB (purple) and SG (pink). The second column shows significant weight-loss-adjusted Spearman’s rho correlations (*P* < 0.05) with gene richness at 12 mo (green), and CAP1 in 12 mo samples for RYGB (purple) and SG (pink). The third column shows Spearman’s rho correlations between BCFA/SCFA at 12 mo and the abundance of terminal genes at 12 mo. White asterisks indicate weight-loss-adjusted *P* < 0.05. **d**, Counts of *ato* at baseline and 12 mo after surgery in participants divided by T2D remission at 12 mo. Lines connect samples from the same individual (the change after surgery covaries with remission; LongDat). Boxes show median and IQR; whiskers specify ±1.5× IQR. **e**, Spearman’s correlation between *ato* counts and BCFA/SCFA at baseline. **f**, Spearman’s correlation between *ato* counts at baseline and CAP1 at 12 months. **g**, MGS that were significantly altered after surgery and contain at least one terminal gene for butyrate production pathways (statistics in Supplementary Tables 7 and 8). MGS are coloured by the encoded terminal genes. *P* values are from false discovery rate-adjusted two-sided Wilcoxon signed-rank tests unless otherwise specified. RYGB, *n* = 39 participants; SG, *n* = 38 participants, each with paired samples at baseline and 12 months. Source data

**Extended Data Fig. 5:**
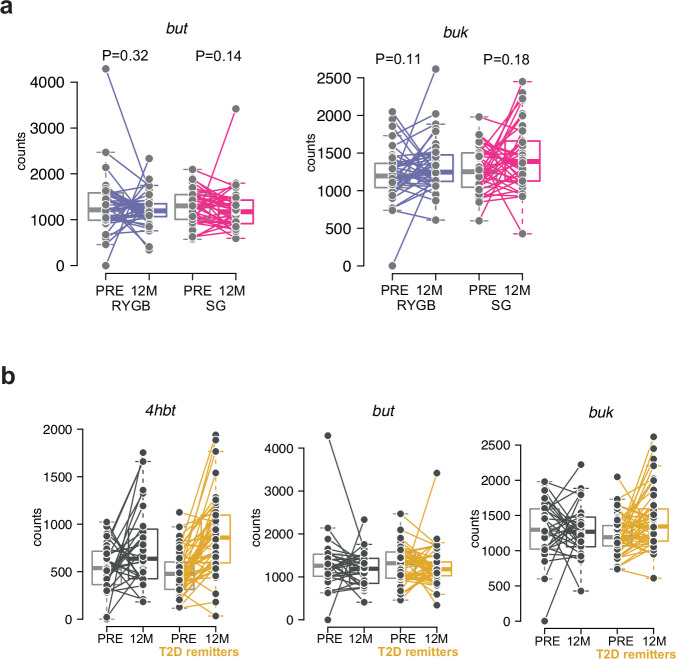
Butyrate terminal genes altered in surgery and linked to remission. **a**. Counts of *but* and *buk* at baseline (PRE) and 12 months after surgery (12M) in RYGB and SG. **b**. Counts of *4hbt*, *but* and *buk* at baseline (PRE) and 12 months after surgery (12M) in participants divided by T2D remission at 12 months. Boxplots show the median (centre), interquartile range (25th–75th percentiles), and whiskers extending to 1.5 × IQR. Dots indicate individual samples and lines connect samples from the same individual. P values are from two-sided Wilcoxon signed-rank tests unless otherwise specified. RYGB, n = 39 participants; SG, n = 38 participants, each with paired samples at baseline and 12 months.

The abundance of *but* and *buk*, the two most prevalent terminal genes, was not significantly different after surgery, but the abundance of *ato* and *4hbt* increased significantly after either surgery type (Fig. 6a–c, Supplementary Table 6 and Extended Data Fig. 5a). Abundance of *buk*, *4hbt* and *ato* at 12 months covaried with CAP1 in both groups, independent of extent of weight loss, and with gene richness and BCFA/SCFA at 12 months (Fig. 6c and Supplementary Table 6). However, *ato* was the only gene that increased in abundance exclusively in those who were in T2D remission (Fig. 6d and Extended Data Fig. 5). Abundance of *ato* at baseline was also significantly associated with BCFA/SCFA at baseline (Fig. 6e) as well as with CAP1 (Fig. 6f). Thus, levels of *ato* before surgery may have predictive value for specific microbiota configurations after bariatric surgery, fermentation capacity and T2D remission.

To identify taxa bearing the terminal genes for butyrate production and that were altered in abundance by surgery, we clustered genes into co-abundant gene clusters and annotated clusters with more than 700 genes (that is, metagenomic species, MGS) that contained the terminal genes for butyrate production. Consistent with increased abundance of *ato* and *4hbt* after surgery, several MGS that increased in abundance after bariatric surgery contained *4hbt*, *ato* or both (Fig. 6g and Supplementary Tables 7 and 8). These MGS were taxonomically annotated as species in *Alistipes*, *Intestinimonas*, *Odoribacter* and *Flavonifractor*, which have been reported to contain *4hbt*^35^. The genera PeH17 and Phil1 in the Christensenellales order were the only MGS bearing *ato* that increased after surgery (Fig. 6g), suggesting that these uncharacterized bacteria might contribute to the increased butyrate production potential after bariatric surgery and are potentially linked to T2D remission.

**Extended Data Fig. 6:**
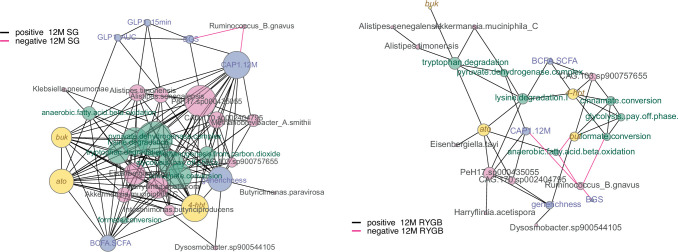
Integrative network following SG and RYGB. Networks of interactions for the 12 months (12M) values of species and GMMs abundances, gene richness, CAP1 and clinical variables associated with T2D remission; SG on the left and RYGB on the right. Circle size indicates number of interactions while black and red lines indicate positive and negative correlations, respectively.

To investigate how gut microbiota configurations 12 months after surgery relate to measures of glucose metabolism important for T2D remission, we performed a network analysis for CAP1, gene richness, species, GMMs and clinical variables associated with T2D remission. This analysis identified more interactions after SG, which again probably reflected the higher heterogeneity in metabolic phenotypes and microbiota composition after this procedure. At 12 months after SG, the abundance of *Alistipes* spp. and PeH17 sp000435055 positively associated with *ato* abundance, CAP1, GLP-1 responses and BGS, while CAP1 and BGS were negatively associated with *R.* *gnavus* (Extended Data Fig. 6). Overall, these results indicate that higher levels of *Alistipes* spp., PeH17 sp000435055 and *ato* are potentially important to achieve a gut microbiota configuration with low levels of *R.* *gnavus* and high BGS after surgery. The positive association between CAP1 and PeH17 sp000435055 and the negative associations between *R.* *gnavus* and both CAP1 and BGS were also observed after RYGB, thus indicating that these interactions may be important for metabolic improvements independent of surgery type.

#### Surgery type-specific microbiome contributions to T2D remission

We next investigated microbiome alterations that covaried with T2D remission at 12 months after each surgery type separately to identify potential surgery-type-specific contributions to remission. To test the contribution to altered fermentation outputs, we also assessed the covariation of these features with BCFA/SCFA.

**Fig. 7:**
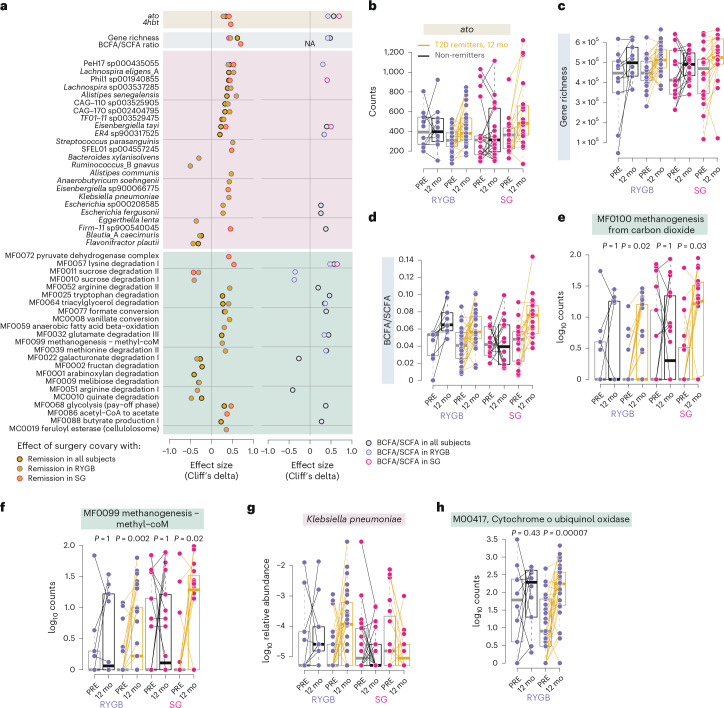
Specific microbiome alterations after RYGB and SG linked to T2D remission. **a**, Left column, effect size (Cliff’s delta) of features with changes in relative abundance after RYGB or SG classified as either ‘entangled with effect’ of remission or ‘reducible to effect’ of remission (yellow points, longDat; see Methods). Right column, effect size (Cliff’s delta) of features with changes in relative abundance after RYGB or SG classified as either ‘entangled with effect’ or ‘reducible to effect’ with change in fermentation ratio (BCFA/SCFA; blue points, longDat; see Methods). Black circles, covarying with BCFA/SCFA ratio in all samples; purple circles, covarying with BCFA/SCFA ratio in RYGB group; pink circles, covarying with BCFA/SCFA ratio in SG group. **b**–**g**, Counts of selected features at baseline and 12 mo after surgery in samples divided by surgery type and T2D remission at 12 mo. Lines connect samples from the same individual. **h**, Abundance of KEGG module M00417 at baseline and 12 mo after surgery in participants with RYGB divided by T2D remission at 12 mo. Boxplots show the median (centre), IQR (25th–75th percentiles) and whiskers extending to 1.5× IQR. *P* values are from unadjusted two-sided Wilcoxon signed-rank tests. RYGB, *n* = 39 participants; SG, *n* = 38 participants, each with paired samples at baseline and 12 months. Additional statistics for panels **a**–**l** are presented in Supplementary Table 9. Source data

Abundance of *ato* and gene richness covaried with remission after both RYGB and SG and levels of *ato* correlated with BCFA/SCFA 12 months after surgery (Fig. 7a–c and Supplementary Table 9), but the increased ratio of BCFA/SCFA covaried only with remission after SG (Fig. 7d). Abundance of PeH17 sp000435055 covaried with remission after either surgery type, while abundance of Phil1 sp001940855, Firm11 sp900540045, *E.* *tayi* and the GMM MF0068 for glycolysis (pay-off phase) covaried with remission only after SG (Fig. 7d). Abundance of the pyruvate dehydrogenase complex (MF0072) and acetyl−CoA to acetate (MF0086) covaried with remission only after SG (Fig. 7a), while the increase in potential for methanogenesis (MF0099) covaried with remission only after RYGB (Fig. 7a). However, targeted analysis showed that methanogenesis increased only in the subgroup with remission after both SG and RYGB (MF0099 and MF0100; Fig. 7e,f). We also found that Proteobacteria such as *Escherichia* and *Klebsiella pneumoniae* covaried with remission after RYGB; Fig. 7a,g). In agreement, vanillate conversion (GMM MC0008) (Fig. 7a), including the KO for the vanillate monooxygenase (*vanB*; K03863), and KOs for the *E.* *coli* respiratory complex cytochrome *o* ubiquinol oxidase (K02297, K02298, K02299, K02300; KEGG module M00417) increased after RYGB in the subgroup with remission (Fig. 7h and Supplementary Table 3), suggesting that increased oxygen-dependent metabolism in the gut is associated with remission specifically in RYGB.

#### Microbiome features can predict 5-year T2D remission

**Fig. 8:**
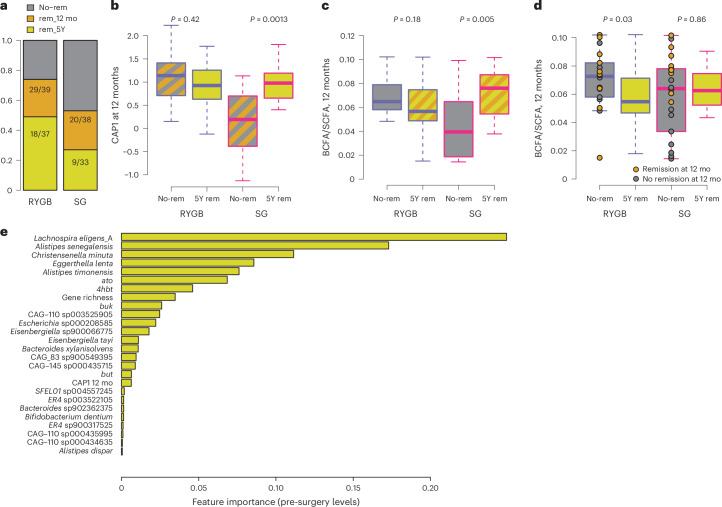
The microbiome shift is predictive of long-term remission in SG. **a**, Percentage of participants with RYGB or SG in remission from T2D at the 12 mo or 5 year (5Y) follow-up. **b**. CAP1 of the faecal microbiota at 12 mo in samples divided by surgery type and T2D remission at 5 year follow-up. **c**. BCFA/SCFA ratio in 12 mo in samples divided by surgery type and T2D remission at 12 mo follow-up. **d**, BCFA/SCFA ratio in 12 mo in samples divided by surgery type and T2D remission at 5 year follow-up. Circles indicate samples from participants who were not in remission at the 5 year follow-up; grey and yellow colours indicate no remission or remission at the 12 month follow-up, respectively. **e**, Bar plots showing features important for predicting 5 year T2D remission from baseline values using XGboost (extreme gradient boosting). Boxplots show the median (centre), IQR (25th–75th percentiles) and whiskers extending to 1.5× IQR. *P* values are from unadjusted two-sided Wilcoxon signed-rank tests. RYGB, *n* = 39 participants; SG, *n* = 38 participants, each with paired samples at baseline and 12 months. Source data

**Extended Data Fig. 7:**
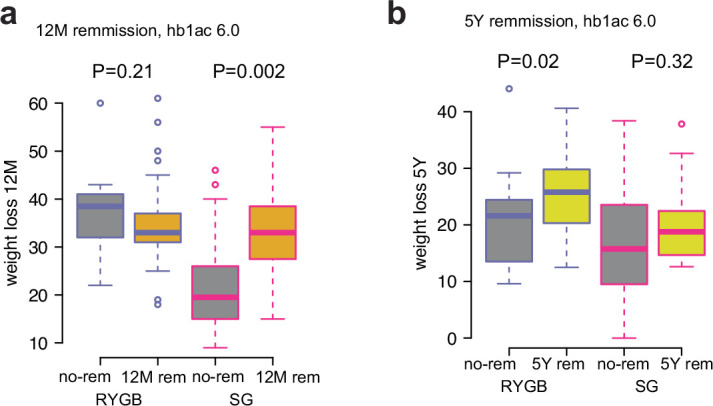
Weight-loss and T2D remission 12 months and 5 years after RYGB and SG. **a**. Weight loss 12 months after surgery (12M) in participants divided by T2D remission at the 12-month follow-up. **b**. Weight loss 5 years after surgery (5Y) in participants divided by T2D remission at the 5-year follow-up. T2D remission was defined as HbA1c ≤ 6.0% without the use of glucose-lowering medications. Boxplots show the median (centre), interquartile range (25th–75th percentiles), and whiskers extending to 1.5×IQR. P values are from two-sided Wilcoxon signed-rank tests unless otherwise specified. RYGB, n = 39 participants; SG, n = 38 participants, each with paired samples at baseline and 12 months. Source data

**Extended Data Fig. 8:**
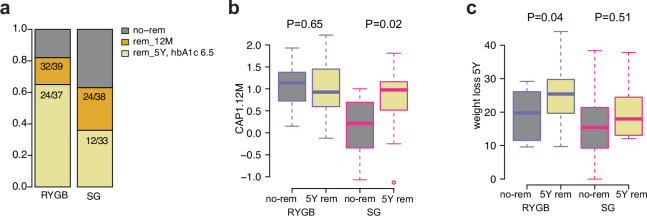
Validation with remission definition of HbA1c ≤ 6.5%. Analysis of data for T2D remission defined as HbA1c ≤ 6.5% without the use of glucose-lowering medications. **a**. Percentage of participants with RYGB or SG in remission from T2D at the 12-month (12 M) or 5-year (5Y) follow-up. **b**. CAP1 of the fecal microbiota at 12 M in samples divided by surgery type and T2D remission at the 5-year follow-up. **c**. Weight loss 5 years after surgery in participants divided by T2D remission at the 5-year follow-up. Boxplots show the median (centre), interquartile range (25th–75th percentiles), and whiskers extending to 1.5 × IQR. P values are from two-sided Wilcoxon signed-rank tests unless otherwise specified. RYGB, n = 39 participants; SG, n = 38 participants, each with paired samples at baseline and 12 months. Source data

Finally, we investigated whether the microbiome alterations observed 12 months after surgery could predict remission at the 5 year follow-up. We found that 49% of the individuals with RYGB and 27% of the individuals with SG were still in remission after 5 years (Fig. 8a). Compared to the 12 month remission status (Fig. 1i), CAP1 was significantly larger for individuals who remained in remission 5 years after SG, but was high in both remitters and non-remitters following RYGB (Fig. 8b). After SG, the higher weight loss observed in remitters after 12 months was no longer observed at the 5 year follow-up (Extended Data Fig. 7). Notably, similar results were observed using the remission definition of HbA1c 6.5% (compared to 6.0% used previously; Extended Data Fig. 8).

After SG, the BCFA/SCFA ratio at 12 months was higher in those in remission at 12 months (Fig. 8c) but not after 5 years (Fig. 8d). This result suggests that BCFA/SCFA is a marker of the current metabolic state of the microbiome and its host and is not applicable to long-term predictions, whereas the microbiome shift over the first year in SG is predictive of long-term T2D remission.

**Extended Data Fig. 9:**
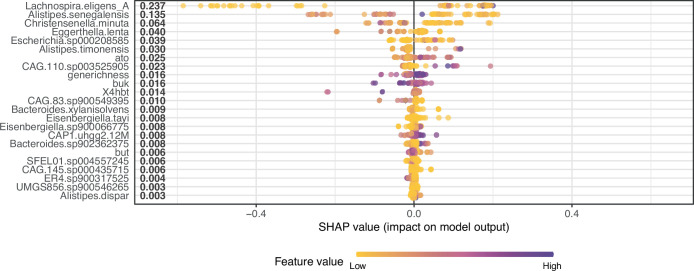
Variables contribution to 5Y remission model. Plot of SHapley Additive exPlanations (SHAP) values for each individual and each variable in the XGBoost model. Variables are ordered by mean absolute SHAP values. Points are colored by features contribution to the model in each individual.

Next, we built a model based on extreme gradient boosting (XGBoost) using CAP1 and baseline values of features associated with 12 month T2D remission to test their importance for long-term prediction of 5 year remission. In addition to gene richness and CAP1, the most predictive features were *L.* *eligens*, *A.* *senegalensis*, *C.* *minuta*, as well as *ato, 4hbt* and *buk* (Fig. 8e and Extended Data Fig. 9).

## Discussion

We identified features of the gut microbiota linked to long-term T2D remission following two types of bariatric surgery by analysing the microbiota from the Oseberg randomized controlled trial^16,17^. All patients lost weight to varying extents, but the major microbiota shift (CAP1) was independent of the extent of weight loss. We show that insulin secretion and GLP-1 responses in individuals with T2D were linked with specific gut microbiota configurations, represented by CAP1. Additionally, baseline abundances of features positively associated with CAP1 were also predictive of 5 year remission. These results support the hypothesis that gut microbiota heterogeneity influences metabolic status and responses to treatments, including bariatric surgery^36^.

Whole-genome metagenomic studies have shown that RYGB and SG alter the gut microbiota with shared as well as procedure-specific effects^20,21,37,38^. Overall, these studies report larger microbiota shifts following RYGB^19,21,38,39^, but had limited power to detect features associated with T2D remission^38^. We observed a more heterogeneous microbiome composition in SG, indicated by the large CAP1 range observed 12 months after this surgical procedure. However, higher CAP1 was associated with greater insulin secretion (BGS) and incretin responses (circulating GLP-1) 12 months after SG and with 5 year T2D remission. Importantly, postoperative dietary intake differences between the surgery groups in this cohort^40^ did not explain this shift. However, these associations explained a small fraction of the variance, consistent with the modest effect sizes observed for individual parameters in microbiome studies.

The ability of the microbiome to attain this configuration after surgery was also linked to baseline glucose metabolism (for example, HbA1c and fasting blood glucose) and gut microbiota gene richness, consistent with previous observations^22^. Furthermore, microbiome features, including Christensenellales and *Alistipes* spp., as well as the *ato* and *4hbt* butyrate production pathways, were associated with diabetes remission, which is consistent with the *4hbt* pathway being causally linked with insulin secretion in response to glucose challenge^13^. After RYGB, a larger and more consistent microbiome shift was observed across participants, resulting in limited variation between individuals and few correlations with metabolic parameters. This suggests that other factors may impact remission after RYGB, including T2D duration^41^.

As reported in previous studies, bariatric surgery increased host mucin degradation^5,21,42^, probably reflecting the increase of *Akkermansia* spp.^43^. However, the abundance of *R.* *gnavus*, a mucolytic proinflammatory bacterium associated with several features of the metabolic syndrome^26^, low gene richness^14^ and inflammatory bowel disease^44^, decreased after surgery. Based on our network analyses, the abundance of *R.* *gnavus* was lower in microbiota with high CAP1 at 12 months after surgery and was negatively associated with insulin secretion (BGS) after both RYGB and SG, suggesting a shared microbiome-associated link with improved β cell function independent of surgery type.

Analysis of the functional potential showed that the gut microbiota at 12 months after surgery was more fermentative in nature, displaying increased potential for production of acetate after SG, propionate after RYGB and butyrate after either surgery. Consistent with the increased fermentative potential, methanogenesis (consuming hydrogen, a main product of anaerobic fermentation) increased after both RYGB and SG. Methanogenesis from carbon dioxide and hydrogen, driven by *M.* *smithii* in the human gut, has been linked to a lean phenotype and metabolic health^11,14,45,46^, as well as fasting^47^ and slow transit time^48^. In our study, methanogenesis was associated with high gene richness and high CAP1, particularly after SG, consistent with previous studies showing a link between *M.* *smithii* and acetate production in communities with high diversity enriched in Christensenellaceae^49^. As increased methanogenesis was associated with T2D remission 12 months after surgery, our results suggest a role for methanogenesis in favouring fermentative metabolism and T2D remission after both RYGB and SG.

In RYGB, we observed increased abundance of *Escherichia* spp. and oxygen-dependent metabolic pathways, including aerobic respiration. These features were linked to high CAP1 and T2D remission specifically in RYGB and occurred together with protein fermentation and anaerobic fatty acid metabolism, consistent with previous reports^5,21^. Both RYGB and SG accelerate gastric emptying and intestinal transit^50^. However, RYGB is characterized by more extensive anatomical rearrangements, influencing bile acid circulation and nutrient delivery^50^ and possibly intestinal redox conditions. The increase of *Escherichia* spp. specifically in RYGB may therefore reflect this more complex ecological restructuring of the gut environment and alterations in luminal redox balance. Furthermore, aerobic respiration may result in energy dissipation, and previous reports have shown an association between increased *E.* *coli* and decreased fat mass after RYGB^8^.

Quantification of fermentation products combined with longitudinal data and functional profiling of the microbiome showed that the potential for SCFA production increased but the faecal levels of these metabolites decreased after surgery. In line with this result, other studies have shown that the functional potential for butyrate production does not correlate with faecal butyrate levels^13^. Our results suggest increased SCFA use after surgery, either by the host, which may be linked to decreased inflammation and increased GLP-1 (refs. ^51,52^), or by microbial cross-feeding, as described for gut bacteria consuming acetate and lactate for butyrate production^53,54^. Accumulation of SCFA in faeces might reflect the current metabolic status of the host, and increased faecal SCFA levels have previously been associated with obesity and features of the metabolic syndrome^55,56^. Increased faecal propionate has been found in obesity^55^ and has been causally linked with increased risk of T2D^13^. We observed that faecal propionate and butyrate decreased only in individuals with T2D remission, driving the increase in the BCFA/SCFA ratio, and BCFA/SCFA was associated with remission in SG. Therefore, measurement of faecal BCFA/SCFA ratios might be useful to follow host–microbiome interactions relevant for metabolic health and T2D. However, faecal concentrations cannot be used to directly infer microbiome fermentation capacity, as SCFA and BCFA levels reflect the combined outcome of microbial production, host absorption and excretion. Dedicated flux studies would be required to directly estimate microbial production.

The species showing strong associations with T2D remission in the full cohort independent of the extent of weight loss were uncharacterized Christensenellales, *L.* *eligens* and *Alistipes* spp., which increased 12 months after surgery and were predictive of 5 year T2D remission, also from baseline. In line with our results, the abundance of these bacteria has been linked with human metabolic health. *L.* *eligens* is a universal human gut bacterium^57^, inducing the production of the anti-inflammatory cytokine IL-10 in vitro^58^. *L.* *eligens* is decreased in atherosclerosis^59^ and is a signature marker decreased in liver cirrhosis, together with *Alistipes* spp.^60^. In a meta-analysis of 8,117 faecal metagenomes from Europe, the USA, Israel and China^57^, *L.* *eligens* and *Alistipes* spp. were negatively associated with T2D, independent of age, sex and metformin medication. Furthermore, *Alistipes* spp. have been associated with insulin sensitivity^61^ and decreased risk of developing T2D over a 15 year follow-up in Finnish individuals^62^. Within Christensenellales, members of the Christensenellaceae family have been associated with host health^63^ and have been linked to healthy ageing^58^; moreover, *C.* *minuta* is decreased in obesity and T2D^46,64^ and is shown to have anti-inflammatory effects in vitro and in mouse models^65^.

This study has some limitations. Our analyses were based on species-level taxonomic resolution, which may mask strain-level functional diversity and limit mechanistic interpretation. Additionally, only participants who provided stool samples were included in the microbiome analyses, and these individuals had slightly lower baseline HbA1c than the full Oseberg cohort. Although this may introduce selection bias at the cohort level, there were no significant metabolic or microbiota differences between the randomized surgery groups within the microbiome subset; therefore, the validity of the comparisons between RYGB and SG is unlikely to be affected.

In conclusion, we identified several gut bacteria predictive of long-term T2D remission after bariatric surgery independent of the extent of weight loss. These bacteria may represent interesting candidates for the development of next-generation probiotics or other microbiome-based interventions targeting T2D. However, as these bacteria are not well characterized or not yet isolated, more work is needed to understand their role in the human gut and the interactions with the host.

## Methods

### Ethical approval

The study protocol^16^ was approved by the Regional Committees for Medical and Health Research Ethics in Norway (2012/1427/REK sør-øst B). Informed consent was obtained from all participants.

### Participant recruitment and clinical data collection

The Oseberg study is a single-centre, triple-blind, randomized controlled trial conducted at Vestfold Hospital Trust (Tønsberg, Norway) (ClinicalTrials.gov registration: NCT01778738)^16^. Patients with severe obesity and T2D who were scheduled for bariatric surgery were screened for eligibility according to the following criteria: age 18 years or older, current body mass index of 33.0 kg m^−2^ or higher with previously verified body mass index of 35.0 kg m^−2^ or higher and T2D (HbA1c of ≥6.5% (48 mmol mol^−1^) or use of antidiabetic medications with HbA1c of ≥6.1% (43 mmol mol^−1^)). Sex was recorded for all participants and considered in the study design. Informed consent was obtained from all participants. Participants did not receive financial compensation.

The main exclusion criteria were previous major abdominal surgery, cancer, severe medical conditions associated with increased risk of complications, drug or alcohol addiction, pregnancy and severe gastro-oesophageal reflux disease. Detailed exclusion criteria can be found in the study protocol^16^. Eligible participants were randomly allocated to either RYGB or SG in a 1:1 ratio; see protocol for description of interventions^16^. A surgeon, not involved in patient follow-up, generated the randomization sequence using a computerized random number generator with a block size of ten. Allocations to personnel and patients were concealed by using sequentially numbered, sealed opaque envelopes. The allocation was revealed to the bariatric surgeon in the operating theatre on the day of surgery, but the blinding was not lifted for the patient or personnel involved in patient follow-up until 1 year after surgery.

**Extended Data Fig. 10:**
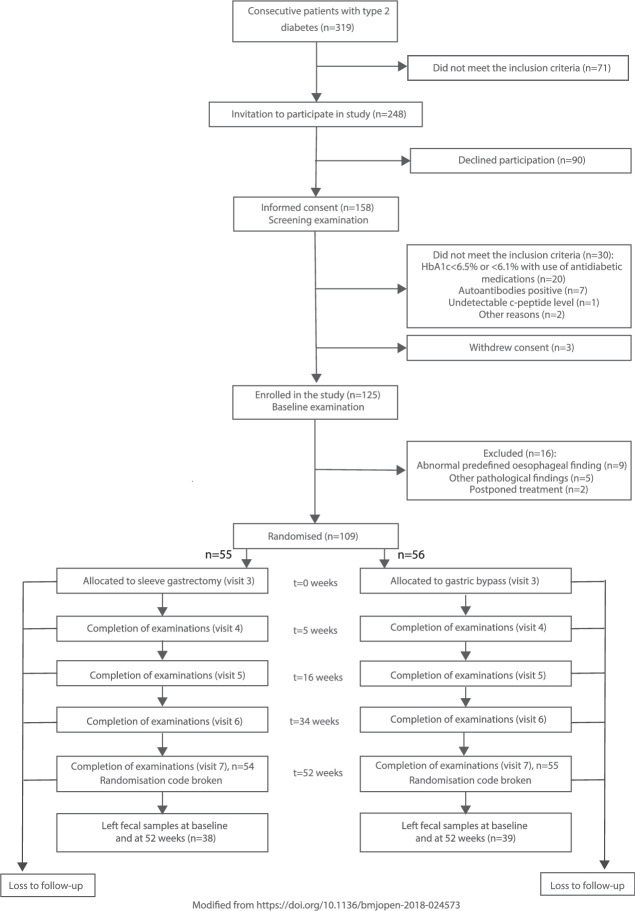
CONSORT diagram. CONSORT diagram of participants in Oseberg which gave fecal samples.

The present study includes 77 patients (39 RYGB and 38 SG) of the 109 originally randomized participants who provided faecal samples both before and 12 months after surgery (Extended Data Fig. 10). The patients who provided faecal samples were on average 8 years older (*P* < 0.001) and had lower HbA1c (7.8% (1.5%) vs 8.9% (1.9%)) than the patients who did not provide faecal samples at both time points (*n* = 32). There were, however, no differences in any other baseline characteristics (Supplementary Table 10). The analyses were not stratified by sex because of the limited sample size.

Dietary habits in the participants were assessed, before and after surgery, using a dietary food frequency questionnaire (FFQ). A registered dietician interviewed all patients individually, at baseline and at the 12 month follow-up, and completed the FFQ together with the patient. The patients and the assessor were blinded to the allocated treatment on both occasions. The FFQ was developed by the Department of Nutrition at the University of Oslo in Norway, and the nutrient intake and energy intake estimated from the FFQ have been validated against doubly labelled water and Acti-Reg^66–68^. Given that energy and micronutrient intake were reduced after surgery, we used macronutrient intakes normalized to energy intake to investigate whether changes in nutrient intake could explain changes in microbiome features.

Data on weight loss and T2D remission status at 5 year follow-up were available for 33 out of 38 participants in the SG group and 37 out of 39 participants in the RYGB group. Two definitions of T2D remission were used: HbA1c ≤ 6.0% (42 mmol l^−1^) or HbA1c < 6.5% (48 mmol l^−1^), both cut-offs without the use of glucose-lowering medication.

### Laboratory analyses

Whole-blood HbA1c was analysed on a Tosoh high-performance liquid chromatography G8 analyser (Tosoh Corporation). Glucose (Vitros 5.1, Ortho-Clinical Diagnostics, until October 2017, and Cobas 8000 analyser, Roche Diagnostics, thereafter), C-peptide and insulin (Cobas 6000 and Cobas e601 analyser, Roche Diagnostics) and GLP-1 (radioimmunoassay specific for the carboxyl terminus of the GLP-1, antibody code no. 89390; RRID:AB_2892195) were analysed in serum obtained before and during the oral glucose tolerance test (OGTT).

### Calculation of insulin secretion and insulin sensitivity

Prehepatic insulin secretion rate (ISR) was calculated from C-peptide concentrations by deconvolution, using the ISEC software programme^69^. We estimated BGS, a measure of OGTT-derived insulin secretion, as the dose–response relationship between ascending post-OGTT glucose levels and ISR values. Time to peak glucose concentration was determined for each individual by plotting the ISR values against the corresponding glucose levels until the time to peak glucose in a cross-correlation analysis^70^. The slope of this linear relationship represents BGS.

To estimate total body insulin sensitivity and the AIRg, we used the Bergman minimal model with MINMOD Millennium software^71^. AIRg is defined as the area under the curve during the first 10 min for serum insulin levels. We also calculated the homoeostasis model assessment (HOMA) indices for insulin sensitivity and secretion using the computer-based HOMA2 Calculator^72^, which incorporates fasting C-peptide and glucose levels. The HOMA2B index estimates steady-state β cell function, while the HOMA2S index estimates steady-state insulin sensitivity, both expressed as percentages relative to a normal reference population.

### Sample collection and metagenome sequencing

The participants were instructed to collect a faecal sample at home using a collection kit. Samples were stored at room temperature, delivered to the clinic within 24 h of collection and stored at −80 °C until extraction.

Total genomic DNA was isolated from 100–150 mg of faeces using a repeated bead-beating method^73^. Samples were placed in Lysing Matrix E tubes (MP Biomedicals) and extracted twice in lysis buffer (4% w/v SDS, 500 mmol l^−1^ NaCl, 50 mmol l^−1^ EDTA, 50 mmol l^−1^ Tris-HCl, pH 8) with bead beating at 5.0 m s^−1^ for 60 s in a FastPrep-24 instrument (MP Biomedicals). After each bead-beating cycle, samples were heated at 85 °C for 5 min and centrifuged at full speed for 5 min at 4 °C. Supernatants from the two extractions were pooled, precipitated with isoproanol and purified using the QIAmp DNA purification kit (QIAGEN). Total DNA was eluted in 100 μl of AE buffer (10 mmol l^−1^ Tris-Cl, 0.5 mmol l^−1^ EDTA, pH 9.0).

A 1 μg sample of total faecal DNA was prepared for sequencing using the TruSeq DNA Nano library preparation kit (Illumina) after random fragmentation using a S220 Focused-ultrasonicator (COVARIS). Libraries were sequenced on a NextSeq500 system (Illumina) as paired-end 150 bp reads.

### Pre-processing and taxonomic and functional annotation of metagenome data

Raw metagenome reads were quality filtered, and human reads were removed using kneaddata 0.10.0 with default settings (https://huttenhower.sph.harvard.edu/kneaddata/). Taxonomy was assigned to high-quality paired-end reads using Kraken2 (ref. ^74^) and the UHGG database (v.2.0)^24^ (https://www.ebi.ac.uk/metagenomics/genome-catalogues/human-gut-v2-0). Classification to species level was performed using Bracken (v.2.6.2)^75^. Gut microbiota composition was analysed using Bray–Curtis dissimilarity. High-quality reads were also aligned to a gene catalogue generated from faecal metagenomes of Swedish subjects with pre-diabetes, T2D and normal glucose tolerance^11^, using Bowtie2 with parameters -U–no-sq–no-head -p 16–very-sensitive. Gene abundance profile was further rarefied to 14.8 million reads per sample. Gene richness was calculated as the number of unique genes in rarefied datasets with 11 million reads per sample. For metagenome functional analysis, genes were annotated using the KEGG database^11,76^, and gene read counts were collated into functional gene families (KOs). Differential abundance of KOs was assessed using DESeq2 (ref. ^77^). KOs were summarized into GMM profiles using Omixer-RPM^29^. Covered genes were also clustered into co-abundant gene clusters using the canopy method^78^. We obtained 429 co-abundant gene clusters with more than 700 genes, defined as MGS. The abundance of MGS across samples was represented by the median abundance in a gene group^78^. Taxonomic annotation of MGS was performed with the Genome Taxonomy Database (GTDB)^79^ using the GTDB toolkit^80^. Targeted analysis of butyrate production potential was performed for five terminal genes in four biosynthetic pathways (*4hbt*, *but*, *atoA/D* and *buk*) using a hidden Markov model^35^ and HMMER search (hmmer.org, v.3.2.1) on amino-acid-translated genes (six frames). Results with a full sequence score of >100 were used. An MGS was assigned butyrate-producing potential if it included at least one complete terminal gene for one of the pathways. The potential for butyrate production pathways in each sample was determined by combining the gene counts annotated to the different terminal genes.

### Measurement of SCFAs and BCFAs

Faecal SCFAs and BCFAs were measured using gas chromatography coupled to mass spectrometry detection. A faecal sample of 25–50 mg was mixed with internal standards, added to glass vials and freeze-dried. All samples were acidified with HCl, and SCFAs were extracted with two rounds of diethyl ether extraction. The organic supernatant was collected, the derivatization agent *N*-*tert*-butyldimethylsilyl-*N*-methyltrifluoroacetamide (Sigma-Aldrich) was added and samples were incubated at 20 °C room temperature overnight. SCFAs were quantified with a 7890A gas chromatograph equipped with a DB-5MS Ultra Inert column (30 m, 0.25 mm, 0.25 µm), coupled to a 5975C mass spectrometer (Agilent Technologies). Isotopically labelled standards of isovaleric acid, propionic acid and sodium salts of acetate, isovalerate and butyrate were obtained from Sigma-Aldrich. Quantification was made using a one-point calibration against the corresponding isotopically labelled internal standard. For each analyte, the most intense ion or fragment was selected for quantification.

### Statistical analysis

This is an exploratory analysis of the participants in the randomized controlled trial who provided faecal samples both before and 12 months after surgery. No a priori power calculations were performed for the analyses. Statistical analyses were performed using the R environment and language^81^. Data distribution was assumed to be normal for variables analysed using parametric tests (Supplementary Table 1), but this was not formally tested. Differential abundance after surgery compared to baseline was assessed using a non-parametric Wilcoxon signed-rank test. Differences between groups were assessed using the Wilcoxon rank-sum test. Tests for explained variance in microbiome composition were performed using permutational MANOVA with 9,999 permutations using adonis2 in vegan 2.7-3 (ref. ^82^). Coefficients for features related to the variance explained were extracted using adonis. For the determination of surgery-constrained variance in composition, capscale in vegan was used. Features in count data (species, KOs, GMMs, butyrate terminal genes) were analysed for change over time and surgery using generalized linear models in LongDat (v.1.1.0)^83^ Analysis was performed using ‘count’ as the data type for dependent variables in longdat_disc. Features were considered significant if the adjusted *P* values (false discovery rate adjustment with the Benjamini–Hochberg procedure) from Wilcoxon signed-rank tests were <0.05 (for species) or <0.1 (for GMMs) and if the change over time did not covary with a change in medication (statin, antihypertensive, proton pump inhibitors). Similarly, we tested whether the change in considered features covaried with changes in T2D status or clinical parameters over time. Effect sizes reported in the figures are features in which changes were classified as either ‘entangled with covariate’ or ‘reducible to covariate’. Feature abundances at the 12 month follow-up were correlated with CAP1 and gene richness at 12 months using Spearman correlation and adjusting for weight loss using the function pcor in the ppcor package^84^. Networks of interacting features at 12 months included Spearman’s rho correlations larger than 0.3 or smaller than −0.2 and false discovery rate-adjusted *P* < 0.1. The model predicting 5 year remission using baseline features, abundances and CAP1 at 12 months was performed using XGboost (extreme gradient boosting), a machine learning method using sequential decision trees. Variables included were baseline abundances of the top abundant T2D remission-associated species and baseline counts of terminal genes for butyrate production. The optimal values for hyperparameters for each outcome were detected by performing a grid search on several possible combinations of different variables. The hyperparameters included the number of trees, learning rate, minimal loss to expand on a leaf node, maximum tree depth and subsample proportion. All other parameters were used at their default values. The package XGBoost (v.1.6.0.1) was used in R (v.4.1.0).

### Reporting summary

Further information on research design is available in the Nature Portfolio Reporting Summary linked to this article.

## Supplementary information

Reporting Summary

Supplementary Tables 1–10 1. Demographic, clinical and dietary characteristics and outcomes at baseline and 12 months after surgery. 2. Species altered in RYGB and SG and relation to covariates and extent of microbiome change. 3. KEGG functions (KO) altered in all samples, RYGB and SG. 4. Gut metabolic modules (GMMs), BCFA/SCFA and gene richness altered in RYGB and SG and relation to covariates and extent of microbiome change. 5. SCFA and BCFA altered in RYGB and SG and relation to remission and extent of microbiome change. 6. Terminal genes in butyrate-producing pathways altered in RYGB and SG and relation to covariates and extent of microbiome change. 7. Metagenomics species with butyrate production pathway annotation. 8. Statistics of metagenomics species with butyrate production pathways altered in bariatric surgery. 9. Features linked to remission of T2D and relation to covariate fermentation products, extent of microbiome change and correlation between features and gene. 10. Statistics for clinical parameters in the full Oseberg cohort (*n* = 109), in participants who collected fecal samples (*n* = 77, analysed in this study) and participants who did not collect fecal samples (*n* = 32).

## Source data

Source Data Figs. 1–8. Data Extended Data Figs. 7 and 8 Statistical source data.

## Data Availability

Whole genome sequencing data have been deposited in the European Nucleotide Archive (ENA) under study accession number PRJEB59861 with public access. Access to clinical data collected from this study, including de-identified individual participant data, will be made available upon email request to J.H. (joran.hjelmeseth@siv.no). Data will be shared with investigators whose proposed use of the data has been approved by the Oseberg Steering Committee and is according to the consent given by the participants and Norwegian laws and legislation. Summarizing source data for figures are provided in Supplementary Tables; individual feature source data for figures are available at Zenodo via 10.5281/zenodo.19203868 (ref. ^85^). Source data are provided with this paper.

## Code availability

Code for figure regeneration is provided at Zenodo via 10.5281/zenodo.19203868 (ref. ^85^).

## References

[1] Carlsson, L. M. S. et al. Life expectancy after bariatric surgery in the Swedish obese subjects study. *N. Engl. J. Med.* **383**, 1535–1543 (2020). 33053284 10.1056/NEJMoa2002449 PMC7580786

[2] Colquitt, J. L., Pickett, K., Loveman, E. & Frampton, G. K. Surgery for weight loss in adults. *Cochrane Database Syst. Rev.* **2014**, CD003641 (2014). 25105982 10.1002/14651858.CD003641.pub4 PMC9028049

[3] Courcoulas, A. P. et al. Seven-year weight trajectories and health outcomes in the Longitudinal Assessment of Bariatric Surgery (LABS) study. *JAMA Surg.* **153**, 427–434 (2018). 29214306 10.1001/jamasurg.2017.5025 PMC6584318

[4] Mingrone, G. et al. Bariatric surgery versus conventional medical therapy for type 2 diabetes. *N. Engl. J. Med.* **366**, 1577–1585 (2012). 22449317 10.1056/NEJMoa1200111

[5] Palleja, A. et al. Roux-en-Y gastric bypass surgery of morbidly obese patients induces swift and persistent changes of the individual gut microbiota. *Genome Med.* **8**, 67 (2016). 27306058 10.1186/s13073-016-0312-1 PMC4908688

[6] Graessler, J. et al. Metagenomic sequencing of the human gut microbiome before and after bariatric surgery in obese patients with type 2 diabetes: correlation with inflammatory and metabolic parameters. *Pharmacogenomics J.* **13**, 514–522 (2013). 23032991 10.1038/tpj.2012.43

[7] Liou, A. P. et al. Conserved shifts in the gut microbiota due to gastric bypass reduce host weight and adiposity. *Sci. Transl. Med.* **5**, 178ra141 (2013). 10.1126/scitranslmed.3005687 PMC3652229 23536013

[8] Furet, J. P. et al. Differential adaptation of human gut microbiota to bariatric surgery-induced weight loss: links with metabolic and low-grade inflammation markers. *Diabetes* **59**, 3049–3057 (2010). 20876719 10.2337/db10-0253 PMC2992765

[9] Tremaroli, V. et al. Roux-en-Y gastric bypass and vertical banded gastroplasty induce long-term changes on the human gut microbiome contributing to fat mass regulation. *Cell Metab.* **22**, 228–238 (2015). 26244932 10.1016/j.cmet.2015.07.009 PMC4537510

[10] Koh, A., De Vadder, F., Kovatcheva-Datchary, P. & Backhed, F. From dietary fiber to host physiology: short-chain fatty acids as key bacterial metabolites. *Cell* **165**, 1332–1345 (2016). 27259147 10.1016/j.cell.2016.05.041

[11] Wu, H. et al. The gut microbiota in prediabetes and diabetes: a population-based cross-sectional study. *Cell Metab.* **32**, 379–390.e3 (2020). 32652044 10.1016/j.cmet.2020.06.011

[12] Chakaroun, R. M., Olsson, L. M. & Backhed, F. The potential of tailoring the gut microbiome to prevent and treat cardiometabolic disease. *Nat. Rev. Cardiol.* **20**, 217–235 (2023). 36241728 10.1038/s41569-022-00771-0

[13] Sanna, S. et al. Causal relationships among the gut microbiome, short-chain fatty acids and metabolic diseases. *Nat. Genet.* **51**, 600–605 (2019). 30778224 10.1038/s41588-019-0350-x PMC6441384

[14] Le Chatelier, E. et al. Richness of human gut microbiome correlates with metabolic markers. *Nature* **500**, 541–546 (2013). 23985870 10.1038/nature12506

[15] Zhao, L. et al. Gut bacteria selectively promoted by dietary fibers alleviate type 2 diabetes. *Science* **359**, 1151–1156 (2018). 29590046 10.1126/science.aao5774

[16] Borgeraas, H. et al. Single-centre, triple-blinded, randomised, 1-year, parallel-group, superiority study to compare the effects of Roux-en-Y gastric bypass and sleeve gastrectomy on remission of type 2 diabetes and beta-cell function in subjects with morbid obesity: a protocol for the Obesity Surgery in Tonsberg (Oseberg) study. *BMJ Open* **9**, e024573 (2019). 31167860 10.1136/bmjopen-2018-024573 PMC6561424

[17] Hofso, D. et al. Gastric bypass versus sleeve gastrectomy in patients with type 2 diabetes (Oseberg): a single-centre, triple-blind, randomised controlled trial. *Lancet Diabetes Endocrinol.* **7**, 912–924 (2019). 31678062 10.1016/S2213-8587(19)30344-4

[18] Wagen Hauge, J. et al. Effect of gastric bypass versus sleeve gastrectomy on the remission of type 2 diabetes, weight loss, and cardiovascular risk factors at 5 years (Oseberg): secondary outcomes of a single-centre, triple-blind, randomised controlled trial. *Lancet Diabetes Endocrinol.* **13**, 397–409 (2025). 40185112 10.1016/S2213-8587(24)00396-6

[19] Morales-Marroquin, E., Hanson, B., Greathouse, L., de la Cruz-Munoz, N. & Messiah, S. E. Comparison of methodological approaches to human gut microbiota changes in response to metabolic and bariatric surgery: a systematic review. *Obes. Rev.* **21**, e13025 (2020). 32249534 10.1111/obr.13025

[20] Paganelli, F. L. et al. Roux-Y gastric bypass and sleeve gastrectomy directly change gut microbiota composition independent of surgery type. *Sci. Rep.* **9**, 10979 (2019). 31358818 10.1038/s41598-019-47332-z PMC6662812

[21] Farin, W. et al. Impact of laparoscopic Roux-en-Y gastric bypass and sleeve gastrectomy on gut microbiota: a metagenomic comparative analysis. *Surg. Obes. Relat. Dis.* **16**, 852–862 (2020). 32360114 10.1016/j.soard.2020.03.014

[22] Aron-Wisnewsky, J. et al. Major microbiota dysbiosis in severe obesity: fate after bariatric surgery. *Gut* **68**, 70–82 (2019). 29899081 10.1136/gutjnl-2018-316103 PMC7143256

[23] Olsson, L. M. et al. Dynamics of the normal gut microbiota: a longitudinal one-year population study in Sweden. *Cell Host Microbe* **30**, 726–739.e3 (2022). 35349787 10.1016/j.chom.2022.03.002

[24] Almeida, A. et al. A unified catalog of 204,938 reference genomes from the human gut microbiome. *Nat. Biotechnol.* **39**, 105–114 (2021). 32690973 10.1038/s41587-020-0603-3 PMC7801254

[25] Forslund, S. K. et al. Combinatorial, additive and dose-dependent drug–microbiome associations. *Nature* **600**, 500–505 (2021). 34880489 10.1038/s41586-021-04177-9

[26] Grahnemo, L. et al. Cross-sectional associations between the gut microbe *Ruminococcus gnavus* and features of the metabolic syndrome. *Lancet Diabetes Endocrinol.* **10**, 481–483 (2022). 35662399 10.1016/S2213-8587(22)00113-9

[27] Pasolli, E. et al. Extensive unexplored human microbiome diversity revealed by over 150,000 genomes from metagenomes spanning age, geography, and lifestyle. *Cell* **176**, 649–662.e20 (2019). 30661755 10.1016/j.cell.2019.01.001 PMC6349461

[28] Amir, I., Bouvet, P., Legeay, C., Gophna, U. & Weinberger, A. *Eisenbergiella tayi*gen. nov., sp. nov., isolated from human blood. *Int. J. Syst. Evol. Microbiol.* **64**, 907–914 (2014). 24282142 10.1099/ijs.0.057331-0

[29] Vieira-Silva, S. et al. Species–function relationships shape ecological properties of the human gut microbiome. *Nat. Microbiol.* **1**, 16088 (2016). 27573110 10.1038/nmicrobiol.2016.88

[30] Falony, G., Vieira-Silva, S. & Raes, J. Microbiology meets big data: the case of gut microbiota-derived trimethylamine. *Annu. Rev. Microbiol.* **69**, 305–321 (2015). 26274026 10.1146/annurev-micro-091014-104422

[31] Campbell, J. W., Morgan-Kiss, R. M. & Cronan, J. E. Jr. A new *Escherichia coli* metabolic competency: growth on fatty acids by a novel anaerobic beta-oxidation pathway. *Mol. Microbiol.* **47**, 793–805 (2003). 12535077 10.1046/j.1365-2958.2003.03341.x

[32] Flamholz, A., Noor, E., Bar-Even, A., Liebermeister, W. & Milo, R. Glycolytic strategy as a tradeoff between energy yield and protein cost. *Proc. Natl Acad. Sci. USA* **110**, 10039–10044 (2013). 23630264 10.1073/pnas.1215283110 PMC3683749

[33] Ilhan, Z. E. et al. Distinctive microbiomes and metabolites linked with weight loss after gastric bypass, but not gastric banding. *ISME J.* **11**, 2047–2058 (2017). 28548658 10.1038/ismej.2017.71 PMC5563958

[34] Louis, P. & Flint, H. J. Formation of propionate and butyrate by the human colonic microbiota. *Environ. Microbiol.* **19**, 29–41 (2017). 27928878 10.1111/1462-2920.13589

[35] Vital, M., Howe, A. C. & Tiedje, J. M. Revealing the bacterial butyrate synthesis pathways by analyzing (meta) genomic data. *Mbio* **5**, e00889 (2014). 24757212 10.1128/mBio.00889-14 PMC3994512

[36] Khan, M. T., Nieuwdorp, M. & Backhed, F. Microbial modulation of insulin sensitivity. *Cell Metab.* **20**, 753–760 (2014). 25176147 10.1016/j.cmet.2014.07.006

[37] Kural, A. et al. Changes in the gut microbiota of morbidly obese patients after laparoscopic sleeve gastrectomy. *Future Microbiol.* **17**, 5–15 (2022). 34877878 10.2217/fmb-2021-0043

[38] Murphy, R. et al. Differential changes in gut microbiota after gastric bypass and sleeve gastrectomy bariatric surgery vary according to diabetes remission. *Obes. Surg.* **27**, 917–925 (2017). 27738970 10.1007/s11695-016-2399-2

[39] Li, J. V. et al. Roux-en-Y gastric bypass-induced bacterial perturbation contributes to altered host–bacterial co-metabolic phenotype. *Microbiome* **9**, 139 (2021). 34127058 10.1186/s40168-021-01086-x PMC8201742

[40] Barstad, L. H. et al. Changes in dietary intake, food tolerance, hedonic hunger, binge eating problems, and gastrointestinal symptoms after sleeve gastrectomy compared with after gastric bypass; 1-year results from the Oseberg study—a randomized controlled trial. *Am. J. Clin. Nutr.* **117**, 586–598 (2023). 36811476 10.1016/j.ajcnut.2022.11.016

[41] Jans, A. et al. Duration of type 2 diabetes and remission rates after bariatric surgery in Sweden 2007–2015: a registry-based cohort study. *PLoS Med.* **16**, e1002985 (2019). 31747392 10.1371/journal.pmed.1002985 PMC6867594

[42] Chen, G. et al. Two bariatric surgical procedures differentially alter the intestinal microbiota in obesity patients. *Obes. Surg.* **30**, 2345–2361 (2020). 32152837 10.1007/s11695-020-04494-4

[43] Glover, J. S., Ticer, T. D. & Engevik, M. A. Characterizing the mucin-degrading capacity of the human gut microbiota. *Sci. Rep.* **12**, 8456 (2022). 35589783 10.1038/s41598-022-11819-z PMC9120202

[44] Henke, M. T. et al. *Ruminococcus gnavus*, a member of the human gut microbiome associated with Crohn’s disease, produces an inflammatory polysaccharide. *Proc. Natl Acad. Sci. USA* **116**, 12672–12677 (2019). 31182571 10.1073/pnas.1904099116 PMC6601261

[45] Olsson, L. M. et al. Gut microbiota of obese subjects with Prader–Willi syndrome is linked to metabolic health. *Gut* **69**, 1229–1238 (2020). 31611297 10.1136/gutjnl-2019-319322 PMC7306984

[46] Goodrich, J. K. et al. Human genetics shape the gut microbiome. *Cell* **159**, 789–799 (2014). 25417156 10.1016/j.cell.2014.09.053 PMC4255478

[47] Sang, X. et al. Dynamics and ecological reassembly of the human gut microbiome and the host metabolome in response to prolonged fasting. *Front. Microbiol.* **14**, 1265425 (2023). 37854337 10.3389/fmicb.2023.1265425 PMC10579591

[48] Roager, H. M. et al. Colonic transit time is related to bacterial metabolism and mucosal turnover in the gut. *Nat. Microbiol.* **1**, 16093 (2016). 27562254 10.1038/nmicrobiol.2016.93

[49] Kumpitsch, C. et al. Reduced B12 uptake and increased gastrointestinal formate are associated with archaeome-mediated breath methane emission in humans. *Microbiome* **9**, 193 (2021). 34560884 10.1186/s40168-021-01130-w PMC8464155

[50] Steenackers, N. et al. Adaptations in gastrointestinal physiology after sleeve gastrectomy and Roux-en-Y gastric bypass. *Lancet Gastroenterol. Hepatol.* **6**, 225–237 (2021). 33581761 10.1016/S2468-1253(20)30302-2

[51] Byndloss, M. X. et al. Microbiota-activated PPAR-γ signaling inhibits dysbiotic Enterobacteriaceae expansion. *Science* **357**, 570–575 (2017). 28798125 10.1126/science.aam9949 PMC5642957

[52] Greiner, T. U. & Backhed, F. Microbial regulation of GLP-1 and L-cell biology. *Mol. Metab.* **5**, 753–758 (2016). 27617198 10.1016/j.molmet.2016.05.012 PMC5004117

[53] Duncan, S. H., Louis, P. & Flint, H. J. Lactate-utilizing bacteria, isolated from human feces, that produce butyrate as a major fermentation product. *Appl. Environ. Microbiol.* **70**, 5810–5817 (2004). 15466518 10.1128/AEM.70.10.5810-5817.2004 PMC522113

[54] Shetty, S. A., Boeren, S., Bui, T. P. N., Smidt, H. & de Vos, W. M. Unravelling lactate-acetate and sugar conversion into butyrate by intestinal *Anaerobutyricum* and *Anaerostipes* species by comparative proteogenomics. *Environ. Microbiol.* **22**, 4863–4875 (2020). 33001550 10.1111/1462-2920.15269 PMC7702098

[55] Schwiertz, A. et al. Microbiota and SCFA in lean and overweight healthy subjects. *Obesity (Silver Spring)* **18**, 190–195 (2010). 19498350 10.1038/oby.2009.167

[56] Teixeira, T. F. et al. Higher level of faecal SCFA in women correlates with metabolic syndrome risk factors. *Br. J. Nutr.* **109**, 914–919 (2013). 23200109 10.1017/S0007114512002723

[57] Mei, Z. et al. Strain-specific gut microbial signatures in type 2 diabetes identified in a cross-cohort analysis of 8,117 metagenomes. *Nat. Med.* **30**, 2265–2276 (2024). 38918632 10.1038/s41591-024-03067-7 PMC11620793

[58] Ghosh, T. S. et al. The gut microbiome as a modulator of healthy ageing. *Nat. Rev. Gastroenterol. Hepatol.* **19**, 565–584 (2022). 35468952 10.1038/s41575-022-00605-x PMC9035980

[59] Jie, Z. et al. The gut microbiome in atherosclerotic cardiovascular disease. *Nat. Commun.* **8**, 845 (2017). 29018189 10.1038/s41467-017-00900-1 PMC5635030

[60] Oh, T. G. et al. A universal gut-microbiome-derived signature predicts cirrhosis. *Cell Metab.* **32**, 878–888.e6 (2020). 32610095 10.1016/j.cmet.2020.06.005 PMC7822714

[61] Takeuchi, T. et al. Gut microbial carbohydrate metabolism contributes to insulin resistance. *Nature* **621**, 389–395 (2023). 37648852 10.1038/s41586-023-06466-x PMC10499599

[62] Ruuskanen, M. O. et al. Gut microbiome composition is predictive of incident type 2 diabetes in a population cohort of 5,572 Finnish adults. *Diabetes Care* **45**, 811–818 (2022). 35100347 10.2337/dc21-2358 PMC9016732

[63] Mancabelli, L. et al. Identification of universal gut microbial biomarkers of common human intestinal diseases by meta-analysis. *FEMS Microbiol. Ecol.* **93**, fix153 (2017). 10.1093/femsec/fix153 29126267

[64] Sun, X. W. et al. *Christensenella*strain resources, genomic/metabolomic profiling, and association with host at species level. *Gut Microbes* **16**, 2347725 (2024). 38722028 10.1080/19490976.2024.2347725 PMC11085954

[65] Kropp, C. et al. *Christensenella minuta* protects and restores intestinal barrier in a colitis mouse model by regulating inflammation. *npj Biofilms Microbiomes* **10**, 88 (2024). 39294159 10.1038/s41522-024-00540-6 PMC11411060

[66] Andersen, L. F. et al. Evaluation of a food frequency questionnaire with weighed records, fatty acids, and alpha-tocopherol in adipose tissue and serum. *Am. J. Epidemiol.* **150**, 75–87 (1999). 10400557 10.1093/oxfordjournals.aje.a009921

[67] Andersen, L. F., Tomten, H., Haggarty, P., Lovo, A. & Hustvedt, B. E. Validation of energy intake estimated from a food frequency questionnaire: a doubly labelled water study. *Eur. J. Clin. Nutr.* **57**, 279–284 (2003). 12571660 10.1038/sj.ejcn.1601519

[68] Carlsen, M. H. et al. Evaluation of energy and dietary intake estimates from a food frequency questionnaire using independent energy expenditure measurement and weighed food records. *Nutr. J.* **9**, 37 (2010). 20843361 10.1186/1475-2891-9-37 PMC2949781

[69] Boston, R. C., Pei, D. & Moate, P. J. A numerical deconvolution method to estimate C-peptide secretion in humans after an intravenous glucose tolerance test. *Metabolism* **58**, 891–900 (2009). 19394979 10.1016/j.metabol.2009.03.003

[70] Byrne, M. M. et al. Insulin secretory abnormalities in subjects with hyperglycemia due to glucokinase mutations. *J. Clin. Invest.* **93**, 1120–1130 (1994). 8132752 10.1172/JCI117064 PMC294056

[71] Boston, R. C. et al. MINMOD Millennium: a computer program to calculate glucose effectiveness and insulin sensitivity from the frequently sampled intravenous glucose tolerance test. *Diabetes Technol. Ther.* **5**, 1003–1015 (2003). 14709204 10.1089/152091503322641060

[72] Wallace, T. M., Levy, J. C. & Matthews, D. R. Use and abuse of HOMA modeling. *Diabetes Care* **27**, 1487–1495 (2004). 15161807 10.2337/diacare.27.6.1487

[73] Salonen, A. et al. Comparative analysis of fecal DNA extraction methods with phylogenetic microarray: effective recovery of bacterial and archaeal DNA using mechanical cell lysis. *J. Microbiol. Methods* **81**, 127–134 (2010). 20171997 10.1016/j.mimet.2010.02.007

[74] Wood, D. E., Lu, J. & Langmead, B. Improved metagenomic analysis with Kraken 2. *Genome Biol.* **20**, 257 (2019). 31779668 10.1186/s13059-019-1891-0 PMC6883579

[75] Lu, J., Breitwieser, F. P., Thielen, P. & Salzberg, S. L. Bracken: estimating species abundance in metagenomics data. *PeerJ Comput. Sci.* **3**, 104 (2017). 10.7717/peerj-cs.104 PMC12016282 40271438

[76] Kanehisa, M. & Goto, S. KEGG: Kyoto Encyclopedia of Genes and Genomes. *Nucleic Acids Res.* **28**, 27–30 (2000). 10592173 10.1093/nar/28.1.27 PMC102409

[77] Love, M. I., Huber, W. & Anders, S. Moderated estimation of fold change and dispersion for RNA-seq data with DESeq2. *Genome Biol.* **15**, 550 (2014). 25516281 10.1186/s13059-014-0550-8 PMC4302049

[78] Nielsen, H. B. et al. Identification and assembly of genomes and genetic elements in complex metagenomic samples without using reference genomes. *Nat. Biotechnol.* **32**, 822–828 (2014). 24997787 10.1038/nbt.2939

[79] Parks, D. H. et al. A complete domain-to-species taxonomy for Bacteria and Archaea. *Nat. Biotechnol.* **38**, 1079–1086 (2020). 32341564 10.1038/s41587-020-0501-8

[80] Chaumeil, P. A., Mussig, A. J., Hugenholtz, P. & Parks, D. H. GTDB-Tk: a toolkit to classify genomes with the Genome Taxonomy Database. *Bioinformatics* **36**, 1925–1927 (2019). 31730192 10.1093/bioinformatics/btz848 PMC7703759

[81] R Core Team. R: A Language And Environment For Statistical Computing (R Foundation for Statistical Computing, 2018).

[82] Oksanen, J. et al. vegan: community ecology package. v. 2.7-3 https://cran.r-project.org/web/packages/vegan/index.html (2015).

[83] Chen, C. Y., Lober, U. & Forslund, S. K. LongDat: an R package for covariate-sensitive longitudinal analysis of high-dimensional data. *Bioinform. Adv.* **3**, vbad063 (2023). 37359720 10.1093/bioadv/vbad063 PMC10284677

[84] Kim, S. ppcor: an R package for a fast calculation to semi-partial correlation coefficients. *Commun. Stat. Appl. Methods* **22**, 665–674 (2015). 26688802 10.5351/CSAM.2015.22.6.665 PMC4681537

[85] Olsson, L. Oseberg microbiome: script for regeneration of figures. *Zenodo*10.5281/zenodo.19203868 (2026).

